# Targeting the JAK/STAT Pathway: A Combined Ligand- and Target-Based Approach

**DOI:** 10.1021/acs.jcim.0c01468

**Published:** 2021-05-17

**Authors:** Maria Galvez-Llompart, Riccardo Ocello, Laura Rullo, Serena Stamatakos, Irene Alessandrini, Riccardo Zanni, Iñaki Tuñón, Andrea Cavalli, Sanzio Candeletti, Matteo Masetti, Patrizia Romualdi, Maurizio Recanatini

**Affiliations:** † Department of Physical Chemistry, University of Valencia, Av. Vicente Estelles s/n, 46100 Burjassot (Valencia), Spain; ‡ Instituto de Tecnología Química (UPV-CSIC) Universidad Politécnica de Valencia Av. Naranjos s/n, 46022 Valencia, Spain; § Department of Pharmacy and Biotechnology, Alma Mater Studiorum-University of Bologna, via Belmeloro 6, 40126 Bologna, Italy; ∥ Italian Institute of Technology (IIT), Via Morego 30, 16163 Genoa, Italy

## Abstract

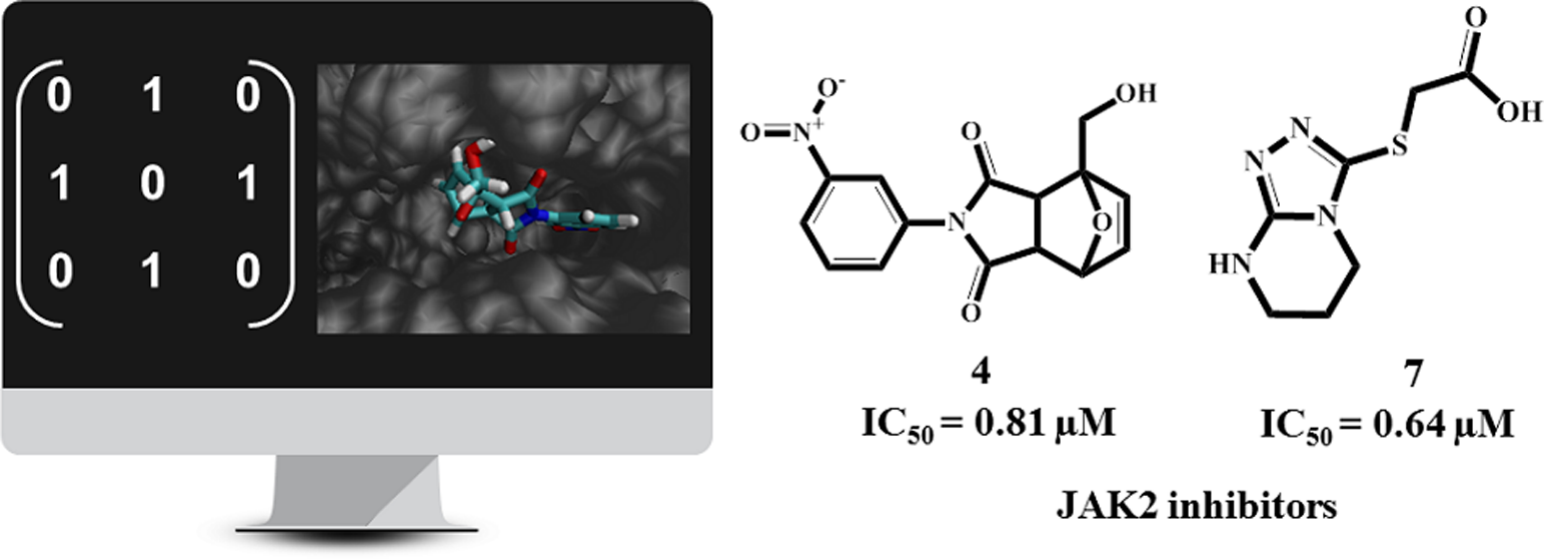

Janus kinases (JAKs) are a family of proinflammatory enzymes able to mediate the immune responses and the inflammatory cascade by modulating multiple cytokine expressions as well as various growth factors. In the present study, the inhibition of the JAK–signal transducer and activator of transcription (STAT) signaling pathway is explored as a potential strategy for treating autoimmune and inflammatory disorders. A computationally driven approach aimed at identifying novel JAK inhibitors based on molecular topology, docking, and molecular dynamics simulations was carried out. For the best candidates selected, the inhibitory activity against JAK2 was evaluated *in vitro*. Two hit compounds with a novel chemical scaffold, **4** (IC_50_ = 0.81 μM) and **7** (IC_50_ = 0.64 μM), showed promising results when compared with the reference drug Tofacitinib (IC_50_ = 0.031 μM).

## 1 Introduction

The Janus kinase (JAK) family of nonreceptor protein tyrosine kinases (PTKs) comprises four mammalian members, JAK1, JAK2, JAK3, and TYK2, that are crucial intracellular components of cytokine and growth factor signaling pathways.^1^ JAK1/2 and TYK2 are ubiquitously expressed, whereas JAK3 is confined to hematopoietic, myeloid, and lymphoid cells.^2^

From a structural standpoint, JAKs share a complex multidomain architecture, unique among PTKs, characterized by seven distinct domains termed the JAK homology (JH1–JH7) domains. A unique feature of JAKs is the presence of two similar but nonidentical domains (JH1 and JH2) at the C-terminus.^3^ While the JH1 domain comprises the highly conserved PTK domain that is critically important for its physiological function, the JH2 domain, also called the pseudokinase domain or kinase-like domain, has no catalytic activity but plays a crucial role in the regulation of the PTK domain.^4^ The JH3–JH4 regions, which share some homology with Src homology 2 domains, have no phosphotyrosine-binding capability and seem to play a structural role in stabilizing the conformation of the JAK FERM domain, which is known to be critical for receptor binding and appears to be essential for the overall regulation of the JAK proteins. Mutations and translocations of the JAK genes, which result in constitutively active JAK proteins, are associated with a variety of hematopoietic malignancies, including autoimmune diseases, myeloproliferative syndromes, leukemia, and lymphomas, as well as cardiovascular diseases.^4^ For example, JAK1 plays an essential role in types I and II interferon signaling and elicits signals from the interleukin-2, interleukin-4, gp130, and class II receptor families. Loss of JAK1 leads to impaired T-cell and B-cell production, a profound defect in interferons. Several JAK1 mutations have been associated with T-cell precursor acute lymphoblastic leukemia and acute myeloid leukemia. A single mutation in the kinase-like domain of JAK2 (V617F), on the other hand, seems to underpin a range of myeloproliferative diseases, such as polycythemia vera, essential thrombocythemia, and chronic idiopathic myelofibrosis. Finally, mature blood cells have a limited life span and are thus continuously renewed in an intricate multistep process. The Janus kinases play an important role in normal hematopoiesis, and their dysregulation can result in a variety of hematological illnesses. These enzymes also play a role in a wide variety of processes including postnatal growth, metabolism, and satiety.

The Janus kinase (JAK)–signal transducer and activator of transcription (STAT) pathway is responsible for the stimulation and production of more than 50 cytokines, many of which are involved in the pathogenesis of autoimmune and inflammatory disorders. As JAK-STAT signaling is required for proper immune function, a loss of the cytokine–JAK-STAT signaling causes immunodeficiency, while an overactivation is related to autoimmune disease and cancer. Cytokine binding induces receptor dimerization and activation of JAK kinase activity, ultimately resulting in activation of STAT proteins. In mammals, the JAK-STAT pathways include four JAKs (JAK1–3 and tyrosine kinase 2, TYK2) and seven STATs (STAT1–5a/b, 6).^5,6^ The possibility of simultaneously blocking a wide array of pathogenic cytokine production via inhibition of the downstream JAK-STAT pathway is becoming increasingly important. Indeed, the US Food and Drug Administration has already approved five JAK inhibitors (Ruxolitinib, Tofacitinib, Baricitinib, Upadacitinib, and Fedratinib) to treat some autoimmune/inflammatory and cancer disorders.^5^ In addition, JAK inhibitors are undergoing clinical trials related to autoimmune and inflammatory diseases.^5^ Taken together, the growing number of diseases in which JAK inhibitors are demonstrating efficacy and the vast pipeline of JAK inhibitors under development make it likely that JAK inhibitors will become crucial in treating autoimmune and inflammatory diseases.^5^

In addition to the above-reported implications related to the discovery of novel JAK inhibitors, we highlight that the regulation of the Janus kinases may also be of importance for the treatment of COVID-19. Indeed, many COVID-19 patients develop acute respiratory distress syndrome (ARDS), which leads to pulmonary edema and lung failure, and also display liver, heart, and kidney damages.^7,8^ These symptoms are associated with a “cytokine storm”, manifesting elevated serum levels of different cytokines (mostly interleukins and interferon). In this context, JAK inhibitors may also play a role in controlling the abnormal cytokine response in severe cases of COVID-19.^9,10^

**Figure 1:**
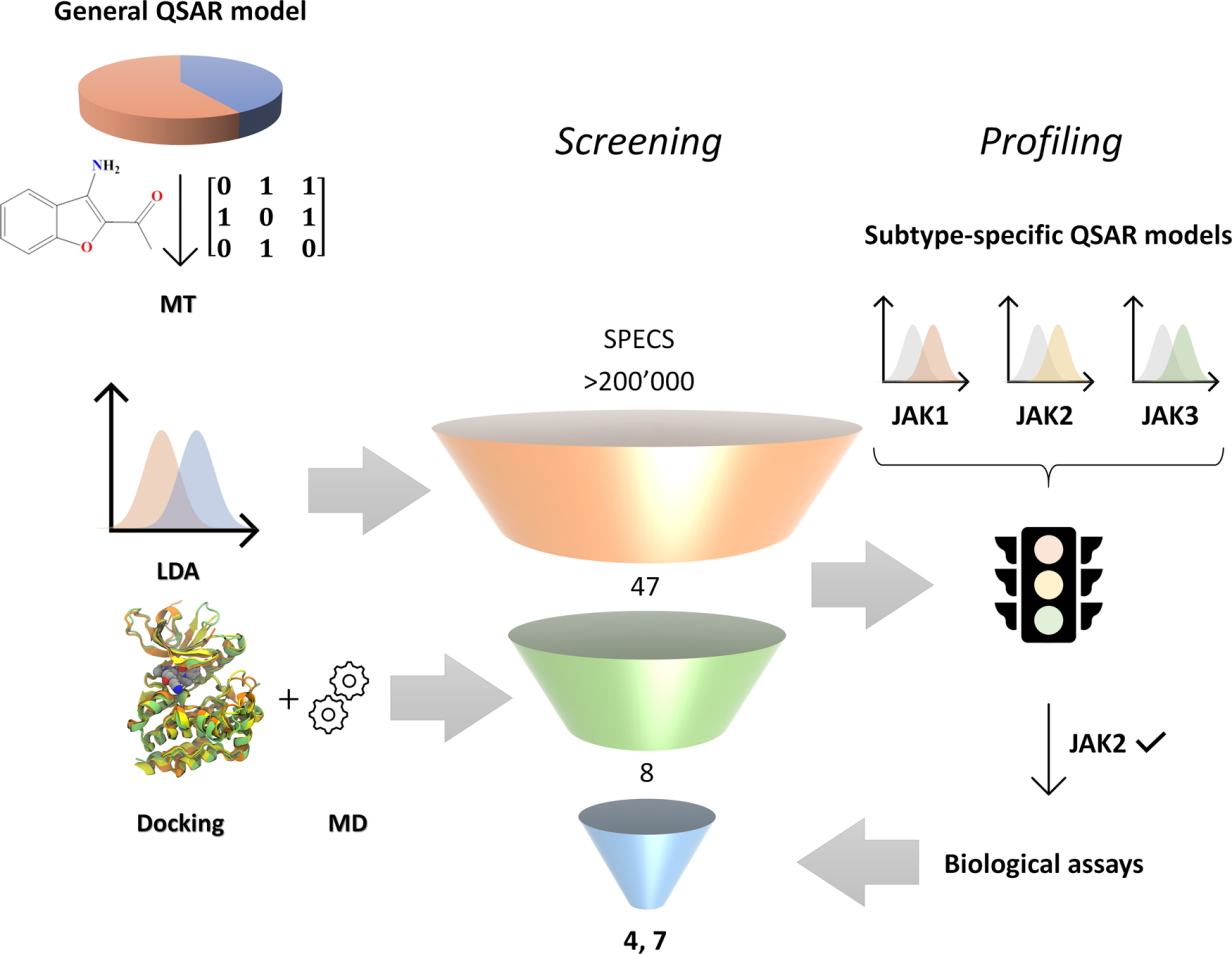
Screening and profiling workflow employed in this work.

Considering the relevance of the JAK-STAT signaling pathway and its dysregulation in several physiopathological processes, there is currently a pressing need to develop novel JAK inhibitors possibly, but not necessarily, showing selectivity toward the distinct subtypes. In the present work, by taking advantage of their complementary features,^11^ we combined ligand- and target-based *in silico* approaches for discovering potential JAK inhibitors (Figure 1). Specifically, a quantitative structure–activity relationship (QSAR) analysis based on molecular topology (MT)^12,13^ and linear discriminant analysis (LDA) was built using a library of known active compounds toward JAKs and decoys. The derived quantitative model was then used for screening the SPECS database and identifying novel putative JAK inhibitors. Finally, the binding mode of the prioritized compounds was characterized via docking calculations and further refined with molecular dynamics (MD) simulations. In parallel, three specific QSAR models were also developed to assess the likelihood of compounds to selectively inhibit the distinct JAK subtypes. The top-ranked compounds were predicted to display a marked (although not exclusive) preference toward the inhibition of JAK2, which was eventually assessed by *in vitro* assays. Even though several studies aimed at identifying novel JAK inhibitors based on molecular docking have been reported in the literature, to the best of our knowledge,^12−17^ this is the first time that QSAR based on MT has been applied for this purpose.

## 2 Results and Discussion

### 2.1 Development and Validation of the QSAR Classification Models

**Figure 2:**
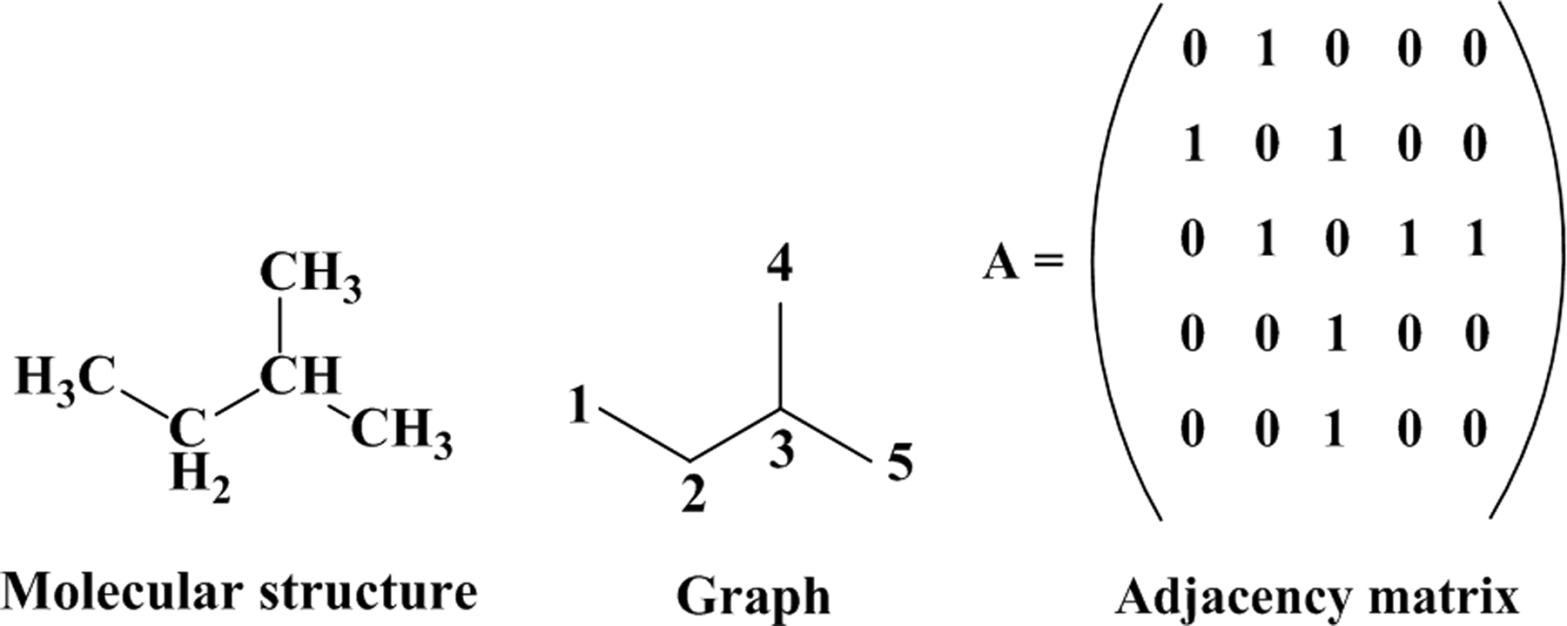
Chemical graph and adjacency matrix of the isopentane.

An *in silico* QSAR strategy based on molecular topology was adopted for discovering novel JAK inhibitors. Generally, QSAR models are developed using the notion of physicochemical descriptors as independent variables. MT represents an alternative paradigm to molecular representation in which molecules are assimilated to a graph, and the resulting adjacency matrix can be used to encode the structure into several mathematical descriptors (often called topological indices; see Figure 2). Notably, MT deals with the connectivity of atoms in molecules and is not related to the geometrical features thereof, such as distances, angles, or tridimensional structure, which is common in other conventional approaches.^18^ MT coupled to LDA allows deriving specific equations (or discriminant functions, DFs) that can be used for predicting if a molecule, not included in the development of the model, will act as an inhibitor or otherwise it will be inactive. During the last decade, MT showed substantial results in drug design, leading to the identification of several new lead molecules in diverse pharmacological and chemical areas.^19^

In this work, four QSAR models were developed. The first model focused on the identification of compounds showing JAK inhibitory activity without preferences regarding the subtypes (hereafter referred to as the general model and mathematically described by the discriminant function DF_gen_). Conversely, the three remaining models were specifically developed for identifying selective inhibitors toward JAK1, JAK2, and JAK3 (referred to as subtype-specific models and described by DF_1_, DF_2_, and DF_3_, respectively).

#### 2.1.1 General Model for Predicting the JAK Inhibitory Activity

The general QSAR model was developed with a training set of known JAK inhibitors and putative inactive molecules (decoys). The model turned out to correctly predict the JAK inhibitory activity for a wide range of structurally unrelated compounds. The discriminant function for this model is reported in eq 1.
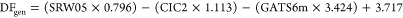
1*N* = 101, Wilks’ Lambda = 0.686, *F* = 14.826, *p* < 0.00001.

**Figure 3:**
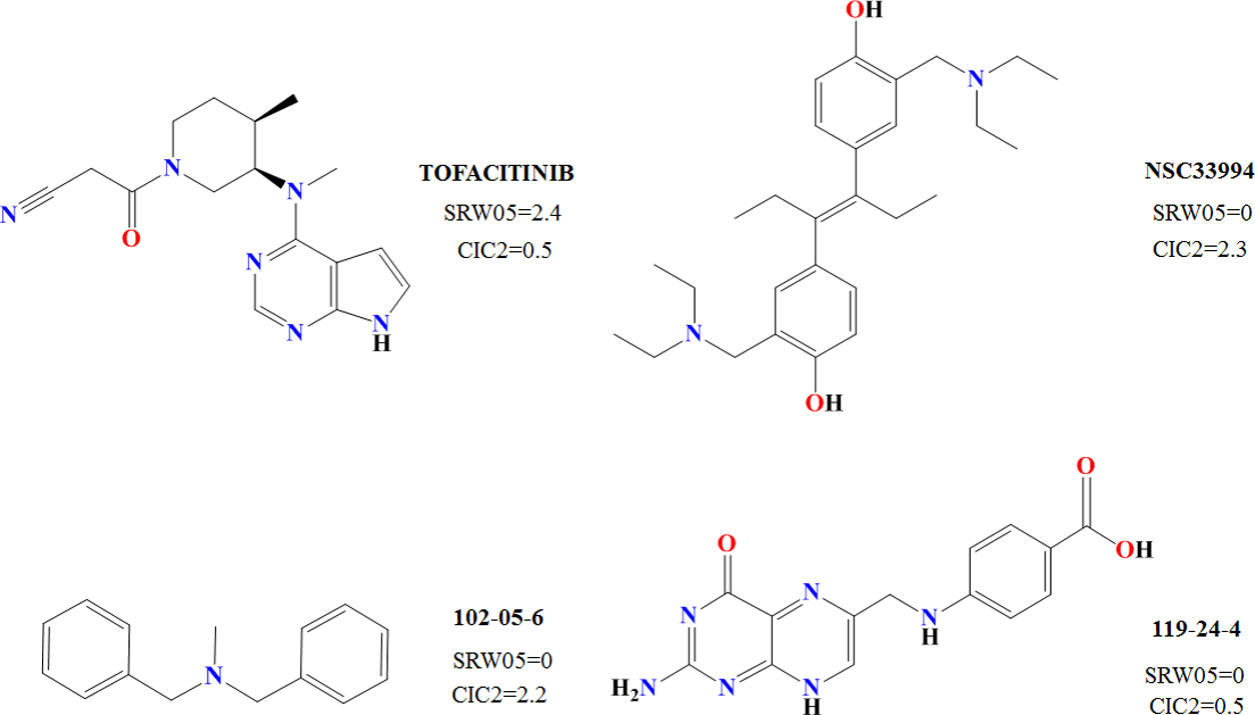
Example of SRW05 and CIC2 values for DF_pan_ for two JAK inhibitors (Tofacitinib and NSC33994) and two decoys (102-05-6 and 119-24-4).

In eq 1, the data set comprised a total of 101 molecules (*N*) including both active and decoys compounds. The Wilks’ statistic for the overall discrimination can take values in the range of 0 (perfect discrimination) to 1 (no discrimination). The Wilks’ Lambda value obtained for this model (0.686) means that DF_gen_ is able to discriminate between active and inactive compounds against JAK inhibition. F statistic, or Fisher–Snedecor *F*, gives information about the significance of the employed variables to explain JAK inhibition. The greater the *F* value, the more significant are the variables to explain the JAK inhibition. Finally, a *p*-value lower than the standard *p* < 0.05 required to reject the null hypothesis, that the observed classification success is no better than that expected by random chance, shows that DF_gen_ is statistically significant. The descriptors employed in DF_gen_ were as follows: self-returning walk count of order 5 (SRW05), Complementary Information Content index – neighborhood symmetry of 2-order (CIC2), and Geary Autocorrelation of lag 6 weighted by mass (GATS6m). SRW05 is considered a walk and path count-type index and is related to the presence of five-membered ring structures. As the descriptor contributes positively to the equation, the presence of five-membered rings is statistically related to the JAK inhibitory activity. Analyzing the values for SRW05 (Table S1 and Figure 3), it can be seen how 31/42 JAK inhibitors have 5-membered rings in their chemical structure, while only 18/59 show this characteristic among decoys.

**Figure 4:**
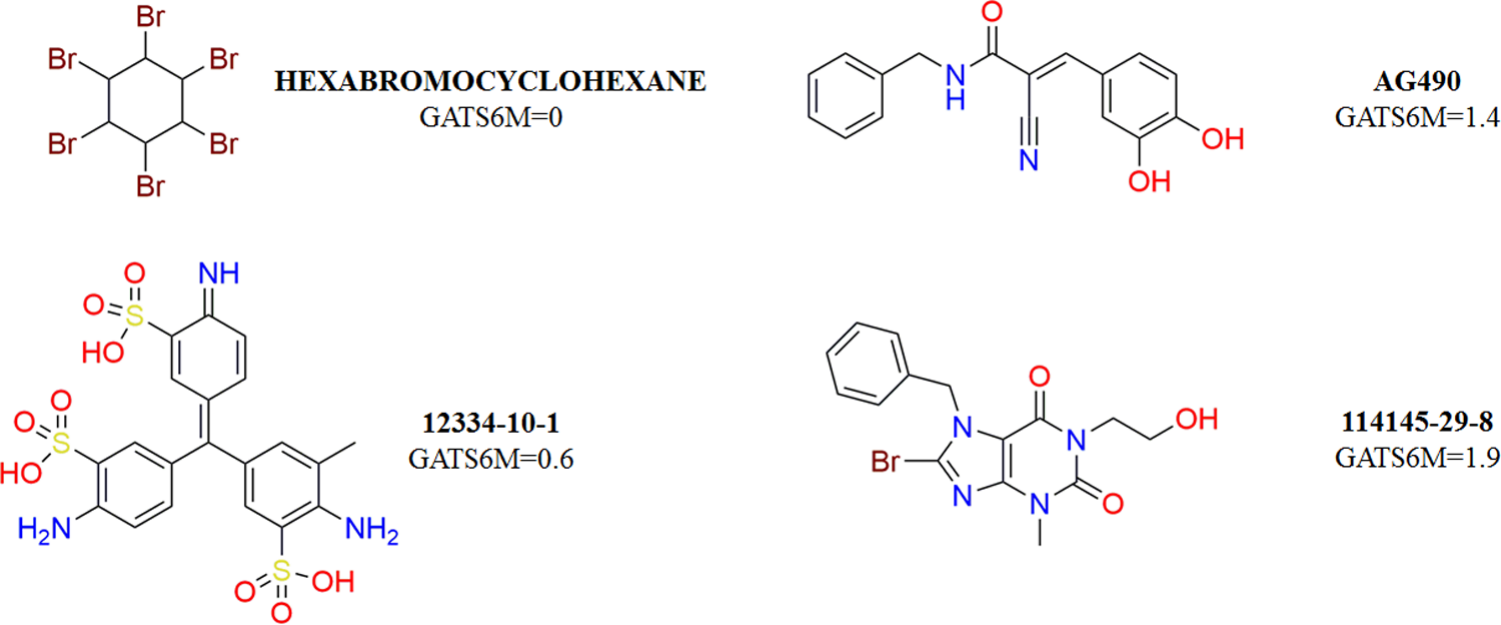
Examples of GATS6m values for two JAK inhibitors (Hexabromocyclohexane and AG490) and two decoys (12334-10-1 and 114145-29-8).

Differently, the CIC2 descriptor is an information-type index, which takes into account the presence of symmetry. Indeed, higher values of this index are related to the presence of symmetric structures (see Table S1 and Figure 3) such as 102-05-06 (CIC2 = 2.227) and NSC33994 (CIC2 = 2.332). Tofacitinib (CIC2 = 0.500) and 119-24-4 (CIC2 = 0.457), however, which do not have symmetric structures, show lower values. As this descriptor contributes negatively to eq 1, it can be hypothesized that structural symmetry may be avoided when searching for JAK inhibitory activity. However, no direct correlation between symmetry and JAK inhibitory activity is demonstrated, as symmetric molecules can be found in both active compounds and decoys (see Figure 3). GATS6m is a 2D autocorrelation descriptor that takes into account the atomic mass for any atom in a structure. This descriptor shows a negative coefficient in eq 1, which indicates that the JAK inhibitory activity indirectly relates to the GATS6m descriptor. Hence, it can be concluded that by increasing the atomic masses at distance 6 between the atoms, the value of the descriptor will increase, causing a reduction in the potential JAK inhibitory activity. However, it can be seen how some molecules show low and high values for GATS6m in either the active or inactive group (see Figure 4). Therefore, this descriptor alone, as for CIC2, cannot discriminate the JAK inhibitory activity among the molecules under study, but at least it gives a useful, general contribution to the analysis.

For any given compound, if the discriminant function DF_gen_ returns values greater than zero, it means that a potential JAK inhibitory activity is expected. Otherwise, the compounds would be labeled as inactive. Table 1 provides information on how DF_gen_ is able to correctly discriminate JAK inhibitors from decoys, yielding an average of correct classification for 77% of the molecules of the training set (see Table S1 in the Supporting Information, SI, for further details). Notice that a random classification should provide no better results than a 50% correctness rate. In addition, DF_gen_ exhibits both sensitivity and specificity, with a correct classification of 79% for the actives and 76% for the inactives.

**Table 1:** Results of the Predictions Obtained with the General Model

	compounds classified as active	compounds classified as inactive	correct classification (%)
Training set			
active group	33	9	79
inactive group	14	45	76
total	47	54	77
Test set			
active group	23	3	88
inactive group	9	24	72
total	31	28	80

**Figure 5:**
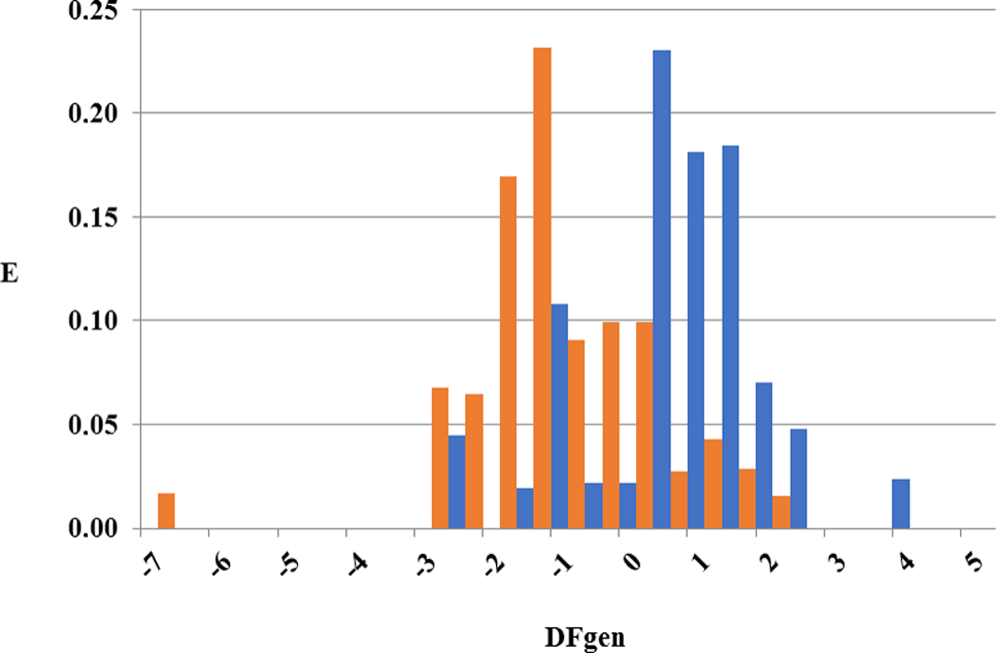
PDD for the general model. Blue bars represent the distribution of JAK inhibitors, and orange bars represent decoys.

Once the model has been derived, it is possible to inspect how active and inactive compounds distribute among the different ranges of the discriminant function equation, determining the region in which the probability of finding active compounds is the maximum. For this purpose, the pharmacological distribution diagram (PDD) can be used. As it can be seen in Figure 5, the majority of known JAK inhibitors tend to peak at values of DF_gen_ > to 0.5, even though a minor density of actives can also be found in the interval [−1: −0.75]. Inactive compounds, on the other hand, show a higher density on DF_gen_ values from −2 to 0.5. Therefore, when this equation will be applied to the search for novel JAK inhibitors, the cutoff value for JAK activity is set for DF_gen_ values between 0.5 and 5 and between −1 and −0.75. Greater and lower DF_gen_ values of 5 and −7, respectively, will be considered as nonclassifiable for this model. Compounds adopting values from −7 to 0.5 (except from the interval from −1 to −0.75) will be considered inactive compounds toward JAK inhibition.

The ability of the general model to correctly classify JAK inhibitors was assessed through an external validation on a test set not employed during the derivation of the model (see Table 1). The external validation revealed that the model is able to identify with high accuracy JAK inhibitors from decoys. Therefore, DF_gen_ seems to be more sensitive than specific (28% of compounds classified as active are false positives, and only 12% of compounds predicted as inactive are false negatives) (see also Table S2 in the SI for further details).

#### 2.1.2 Specific QSAR Models for Predicting Selective Inhibitory Activity toward Each JAK Subtype

The general model described in the previous section provides useful insights regarding the JAK inhibitory activity of molecules; however, more information about which JAK subtype is actually targeted is of great importance. Therefore, three additional QSAR models specific for each JAK subtype were derived. The discriminant functions DF_1-3_ of the subtype-specific models are reported in Table 2. The descriptors employed in eqs 2–4 belong to different families of topological and topo-chemical descriptors, such as (i) walk and path counts (PCD: difference between multiple path count and path count); (ii) 2D autocorrelation descriptors, like the Moran autocorrelation of lag 5 weighted by the ionization potential (MATS5i), the Geary autocorrelation of lag 4 weighted by the Sanderson electronegativity (GATS4e), the Geary autocorrelation of lag 8 weighted by mass (GATS8m), and the Geary autocorrelation of lag 5 weighted by the Sanderson electronegativity (GATS5e); (iii) edge adjacency indices such as Eig05_AEA(dm) (eigenvalue n. 5 from the augmented edge adjacency matrix weighted by the dipole moment); (iv) P_VSA-like descriptors such as P_VSA_LogP_5 (P_VSA-like on Log *P*, bin 5); and (v) topological charge indices such as JGI8 (mean topological charge index of order 8).

**Table 2:** Models Predicting JAK1, JAK2, and JAK3 Inhibitory Activity and Statistical Parameters

model	eq. no.	*N*	λ	*F*	*p* <
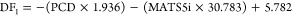 2	eq 2	29	0.462	15.155	0.00001
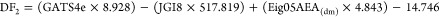 3	eq 3	29	0.490	9.009	0.0003
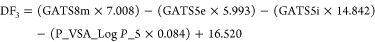 4	eq 4	29	0.462	6.706	0.0010

The different descriptors employed in the distinct models provide useful information regarding the topological and/or physicochemical features that are required for the specific inhibitory activity toward JAK1, JAK2, or JAK3 subtypes. For example, it is possible to see how JAK1 inhibitors present negative values for the MATS5i index and PCD index values lower than 4 (see also Table S3 for the compounds Baricitinib, Ruxolitinib, Itacitinib, Solcitinib, PF-04965842, Oclacitinib, Momelotinib, and Filgotinib). Since both indices contribute negatively to the equation, the fact that they adopt small unsigned values is correlated with favoring the inhibitory activity against JAK1. Regarding the inhibitory activity against the JAK2 subtype, it is observed that compounds with values of the JGI8 index < 0.01 always present JAK2 activity (see Table S4, compounds Hexabromocyclohexane, Ruxolitinib, XL019, Pacritinib, AT9283, Momelotinib, Tofacitinib, Cerdulatinib, WP1066, Filgotinib, Go6976, Gandotinib). Conversely, the inhibitory activity toward JAK2 is observed for JGI8 index values >0.01 only when the descriptor Eig05_AEA (dm) adopts values greater than 2.5 (see Table S4, compounds baricitinib, NVP -BSK805, CEP33779, TG101209, BMS-911543, and AZ-960), in agreement with eq 3. Concerning eq 4 (Tables 2 and S5), we can see that except for the GATS8m descriptor that contributes positively to the inhibitory activity against JAK3, in general, the higher the value the remaining descriptors adopt, the lower is the ability to inhibit this JAK subtype.

In Table 3, we summarize the performance of the three specific models. As it can be seen, the correct classification for the three predictive models against different JAK subtypes presents an average value higher than or equal to 83%. In addition, it should be noted that all models are more specific than sensitive, a feature that makes it difficult to select false assets when using these models for database screening.

**Table 3:** Percentage of Correct Classifications for the Subtype-Specific Models

	percent of correct classification		
	DF_1_	DF_2_	DF_3_
active group compounds	73	90	82
inactive group compounds	89	90	94
total	83	90	89
internal validation^a^	77	83	87

a Average value.

**Figure 6:**
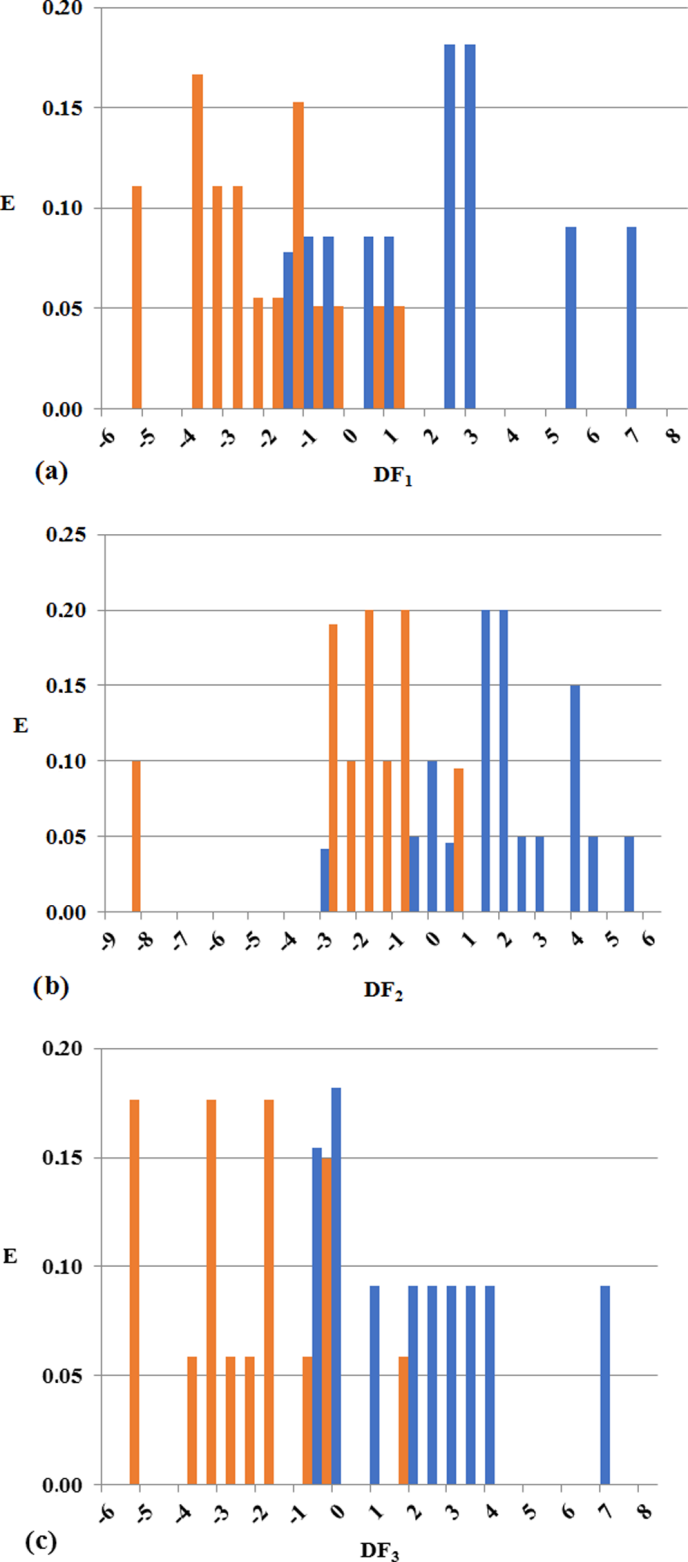
PDD for the subtype-specific models: DF_1_, DF_2_, and DF_3_ in panels (a), (b), and (c), respectively. Black bars represent the distribution of JAK inhibitors, and white bars represent decoys.

An internal validation process, “leave some out” cross-validation, has been performed on the models. A maximum difference of 7% between the values obtained by the models and the internal validation can be observed (Tables 3 and S6, S7, and S8), showing that all models developed for the prediction of inhibitory activity against the different JAK subtypes are robust and predictive. In Figure 6, the PDDs for the subtype-specific models are shown. As it could be seen, JAK1 inhibitors are mainly present in DF_1_ ranges spanning from −1 to 8, while JAK2 inhibitors are found at values ranging from −0.5 to 6 on DF_2_. Finally, JAK3 inhibitors are present mostly in ranges of DF_3_ going from −0.5 to 7.5.

### 2.2 Virtual Screening of a Commercial Database

The general QSAR model for JAK inhibition was used to perform a ligand-based virtual screening on the Specs database of commercial compounds. Using the cutoff values of DF_gen_ inferred from the PDD shown in Figure 4, we selected 47 compounds that were further profiled by the subtype-specific models for assessing their preferential ability to inhibit the activities of JAK1, JAK2, or JAK3. In Table 4, the prioritized compounds and their classification according to the subtype-specific models are reported.

**Table 4:** Discriminant Function Values Predicted by the Different Models for All 47 Selected Compounds^b^^c^

	JAK model	JAK1 model		JAK2 model		JAK3 model	
compound	DF_gen_	DF_1_	class.	DF_2_	class.	DF_3_	class.
**AA-516/30011028**	**1.370**	**4.408**	**JAK1**	**5.096**	**JAK2**	**–7.554**	
AB-323/13887443	0.996	4.940	JAK1	–2.528		–3.924	
AC-907/34131030	1.729	1.524	JAK1^a^	3.415	JAK2	–5.154	
AE-848/34779061	1.682	1.830	JAK1^a^	0.956		–5.407	
AF-399/13277002	2.313	1.661	JAK1^a^	–9.474		34.181	N.C.
AF-399/13426006	1.013	8.821	N.C.	6.795	N.C.	–6.897	
AF-399/15031149	1.546	0.951	JAK1^a^	–0.561		–5.680	
AF-399/15032375	0.981	5.213	JAK1	–0.973		–7.423	
AF-399/33696009	1.977	5.731	JAK1	2.553	JAK2	–3.293	
AF-399/37297037	1.454	–2.344		–2.130		–0.599	
AF-399/41668884	1.270	6.604	JAK1	2.890	JAK2	–7.146	
AF-399/41945530	1.875	0.214	JAK1^a^	2.056	JAK2	–2.432	
AF-399/42056988	0.978	2.044	JAK1^a^	5.767	JAK2	4.314	JAK3
**AF-399/42100326**	**1.649**	**4.054**	**JAK1**	**2.115**	**JAK2**	**–4.967**	
AF-399/42762404	1.901	5.191	JAK1	3.680	JAK2	–8.082	
AG-205/11444099	0.921	2.650	JAK1	6.925	N.C.	7.388	JAK3
AG-205/11674118	0.993	1.653	JAK1^a^	2.922	JAK2	7.297	JAK3
**AG-205/12010072**	**0.954**	**4.327**	**JAK1**	**1.075**	**JAK2**	**–3.811**	
AG-205/14250132	0.508	1.765	JAK1^a^	–3.150		–4.043	
AG-205/14673025	1.414	1.456	JAK1^a^	2.119	JAK2	–0.373	
AG-401/02041003	1.810	–3.699		–5.515		3.404	JAK3
**AG-670/13619018**	**-0.978**	**3.426**	**JAK1**	**3.325**	**JAK2**	**–0.314**	
AG-690/36926024	2.249	–2.545		–0.516	JAK2	4.618	JAK3
AH-357/03329001	0.530	0.563	JAK1^a^	0.175	JAK2	–5.608	
**AK-778/43206447**	**2.346**	**0.171**	**JAK1**^a^	**5.559**	**JAK2**	**–0.863**	
AK-968/15359231	2.103	3.603	JAK1	3.093	JAK2	–4.128	
AM-807/37225018	0.646	11.083	N.C.	1.879	JAK2	–16.247	
AN-329/11658808	2.503	2.659	JAK1	4.496	JAK2	0.445	JAK3
AN-329/41717385	–1.097	7.420	JAK1	3.673	JAK2	–5.092	
AN-584/40652663	2.879	7.470	JAK1	7.042	N.C.	5.262	JAK3
AN-584/43492329	1.641	3.542	JAK1	3.279	JAK2	6.518	JAK3
AN-988/41531688	0.663	–1.129	JAK1^a^	4.160	JAK2	–3.345	
AO-365/43473564	1.559	4.322	JAK1	–0.516	JAK2	5.543	JAK3
**AO-476/41610187**	**1.340**	**0.351**	**JAK1**^a^	**–9.178**		**3.540**	**JAK3**
AO-476/43250148	1.253	5.967	JAK1	–2.347		0.294	JAK3
AO-476/43250150	2.215	6.816	JAK1	–1.103		–2.306	
AO-476/43250160	1.120	6.705	JAK1	–1.464		-3.291	
**AO-476/43417077**	**1.690**	**–1.031**	**JAK1**^a^	**3.833**	**JAK2**	**1.214**	**JAK3**
AP-064/42049177	0.803	–6.558		–0.021	JAK2	1.564	JAK3
AP-501/43286814	1.120	–0.082	JAK1^a^	1.093	JAK2	–4.604	
**AQ-405/42300191**	**0.548**	**0.990**	**JAK1**^a^	**5.571**	**JAK2**	**–2.172**	
AQ-432/43399984	0.528	5.710	JAK1	3.595	JAK2	–5.423	
AQ-432/43400108	1.617	2.557	JAK1	4.888	JAK2	0.677	JAK3
AQ-432/43400219	0.862	0.113	JAK1^a^	3.845	JAK2	2.936	JAK3
AQ-432/43400304	0.772	5.631	JAK1	3.870	JAK2	–5.792	
AQ-432/43400319	1.602	2.562	JAK1	4.251	JAK2	3.808	JAK3
AT-417/43503979	0.903	3.253	JAK1	8.632	N.C.	20.232	N.C.

a Overlapping zone with other JAK inhibitor subtypes, nonabsolutely sure being correctly classified by this model.

b N.C., not classifiable by this model, out of range of the applicability domain.

c Bold: *in vitro* tested.

### 2.3 Molecular Docking

**Figure 7:**
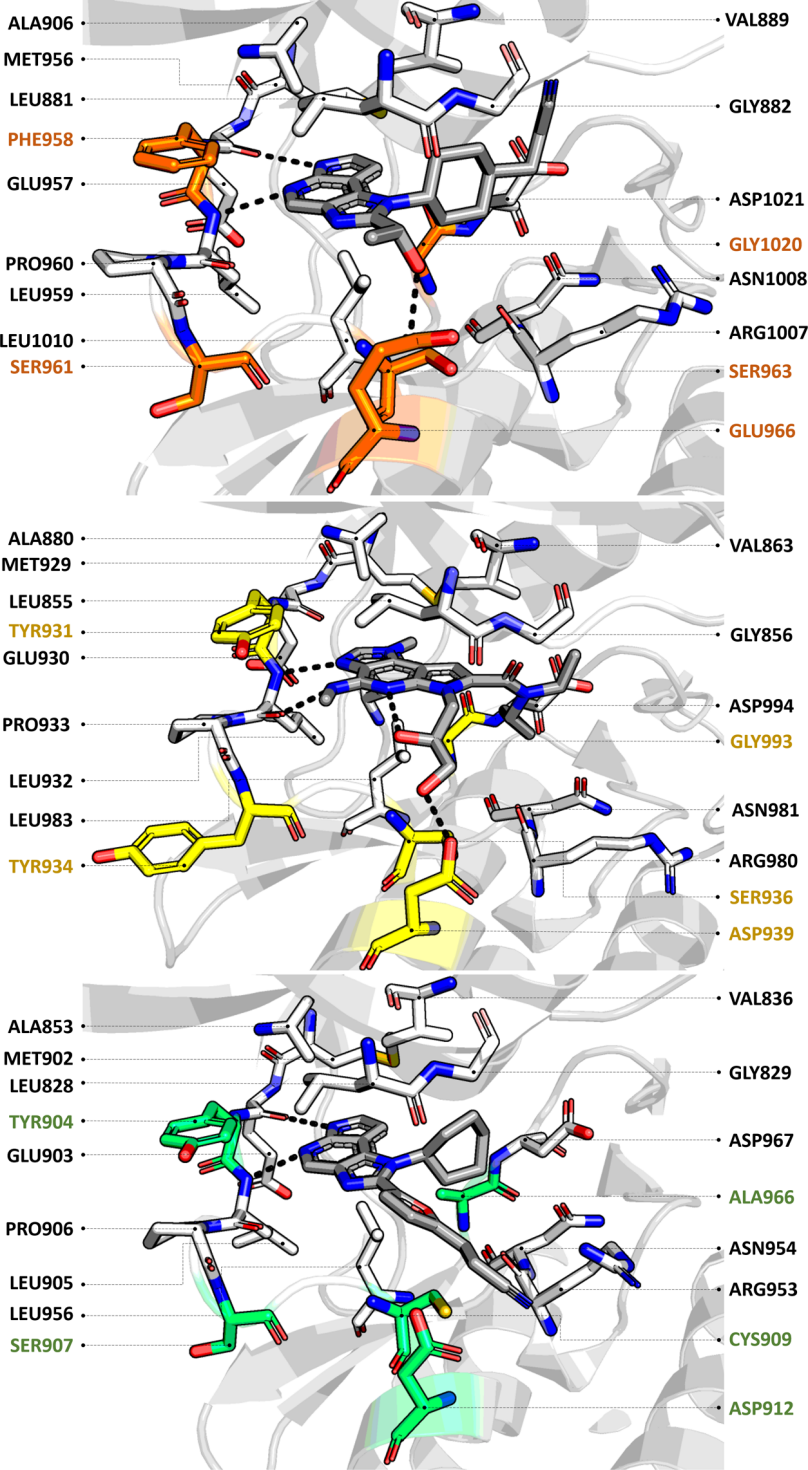
Representation of the binding sites of the JAK1, JAK2, and JAK3 structures in complex with the native ligands that were selected for the docking calculations (PDB-ID: 4IVD, 5CF6, and 6GLA, respectively).^20−22^ The conserved residues are represented as sticks with carbon atoms colored in white, while specific amino acids are differently colored (orange, yellow, and green for JAK1, JAK2, and JAK3, respectively). Hydrogen bonds are explicitly reported as black dots.

To further narrow down the number of potential inhibitors, we adopted the target-based approach of molecular docking. Since the conformational plasticity of the target is only partially taken into account in docking calculations, the results of these methodologies are often very sensitive to the quality of the input structure. Given that a total number of 159 crystallographic structures of the different JAK subtypes were found in the Protein Data Bank (PDB), we set up a simple procedure to choose the most suited protein structures on which performing the docking calculations (see Section 4.2 for details). A cross-docking exercise was then carried out to validate the ability of the docking protocol to reproduce the available experimental complexes and to find out the structures endowed with the better propensity to reproduce not only the native binding mode but also the one displayed by other structures (Figure S1). As a result of the cross-docking, we selected the PDB-IDs 4IVD,^20^5CF6,^21^ and 6GLA(22) as representatives of JAK1, JAK2, and JAK3 subtypes. In Figure 7, we report the main interactions established by the native compounds cocrystallized with the structures chosen to represent the three JAK subtypes.

Once determined the reference crystal structures for JAK1, JAK2, and JAK3, a molecular docking study was performed either for reference JAK inhibitors (Table 5) or for the compounds selected by the general QSAR model (Table S9).

**Table 5:** Docking Score for Known JAK Inhibitors and 4IVD, 5CF6, and 6GLA as Target Proteins

compound	docking score JAK1 (PDB-ID: 4IVD)	docking score JAK2 (PDB-ID: 5CF6)	docking score JAK3 (PDB-ID: 6GLA)
AT9283		**–**6.14^a^	**–**6.95
AZ-960		**–**8.23	
AZD1480			**–**8.63
Baricitinib	**–**8.97	**–**9.49	
BMS-911543		**–**9.68	
CEP33779		**–**9.27	
Cerdulatinib	**–**6.28	**–**8.38	**–**8.28
Decernotinib			**–**9.62
Filgotinib	**–**7.29	**–**9.62	**–**8.85
FLLL32		**–**5.35	
Gandotinib		**–**8.94	
Go6976		**–**8.28	
Hexabromocyclohexane		**–**2.93	
Itacitinib	**–**8.88		
JANEX-1			**–**8.43
Momelotinib	**–**8.84	**–**7.63	
NVP-BSK805		**–**10.05	
Oclacitinib	**–**9.28		
Pacritinib		**–**6.84	
PF-04965842	**–**8.61	**–**7.96	
PF 06551600 malonate			**–**8.97
Ruxolitinib	**–**8.75	**–**9.27	
Solcitinib	**–**8.43		
TG101209		**–**6.01	
**Tofacitinib**	**–9.31**	**–8.50**	**–8.08**
WHI-P154			**–**7.19
WHI-P97			**–**6.60
WP1066		**–**6.15	
XL019		**–**9.30	
ZM39923 hydrochloride	–6.92		**–**6.30

a kcal/mol.

b Bold: reference drug.

As can be seen in Table 5, just Cerdulatinib, Filgotinib, and Tofacitinib show favorable interactions with all three JAK subtypes. Of these drugs, the one that presents a better average docking score value for the different JAKs is Tofacitinib (docking scores of −9.31 kcal/mol JAK1, −8.50 kcal/mol JAK2, and −8.08 kcal/mol JAK3), so this compound will be taken as the reference drug.

**Figure 8:**
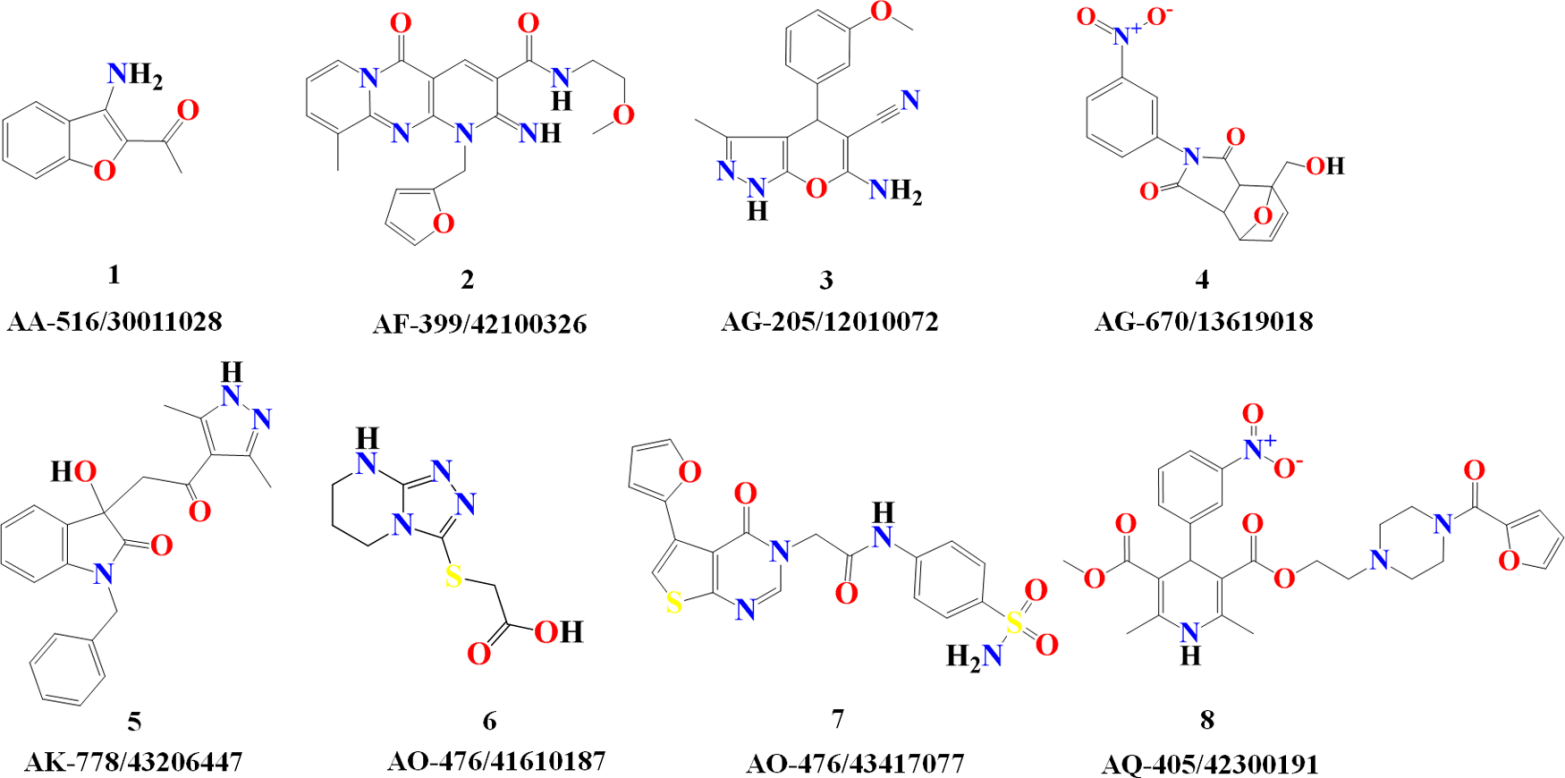
Chemical representation and codification for the eight selected compounds as potential JAK inhibitors.

In Table S9, the values of the docking score for the compounds selected by the general QSAR model are reported. To select the best candidates to be tested *in vitro*, the following criterion was applied: only molecules with docking score equal to or less than **–**7 kcal/mol at least for one JAK subtype were considered. As can be seen in Table S9, 23 compounds from a total of 47 fulfill such a requirement. Of these 23 compounds, based on their commercial availability and price, a final selection of eight compounds was made to be further tested *in vitro* (Figure 8 and Table 6).

**Table 6:** Docking Score for the Prioritized Compounds against Each JAK Subtype^c^

	docking score (kcal/mol)		
compound	JAK1 (PDB-ID: 4IVD)	JAK2 (PDB-ID: 5CF6)	JAK3 (PDB-ID: 6GLA)
**Tofacitinib**	**–**9.31^a^	–8.50	–8.08
**1**	–7.06	–7.61	–8.00
**2**	–6.82	**–9.46**	–6.96
**3**	–8.24	–7.31	–7.89
**4**	–6.81	–8.17	–7.26
**5**	**–9.05**	–7.11	–6.90
**6**	–8.14	–9.03	**–8.32**
**7**	–5.34	–7.34	–6.88
**8**	–8.13	–6.28	–5.73

a kcal/mol.

b Underlined: docking score from the reference drug.

c Bold: top docking score for each JAK subtype under analysis.

**Figure 9:**
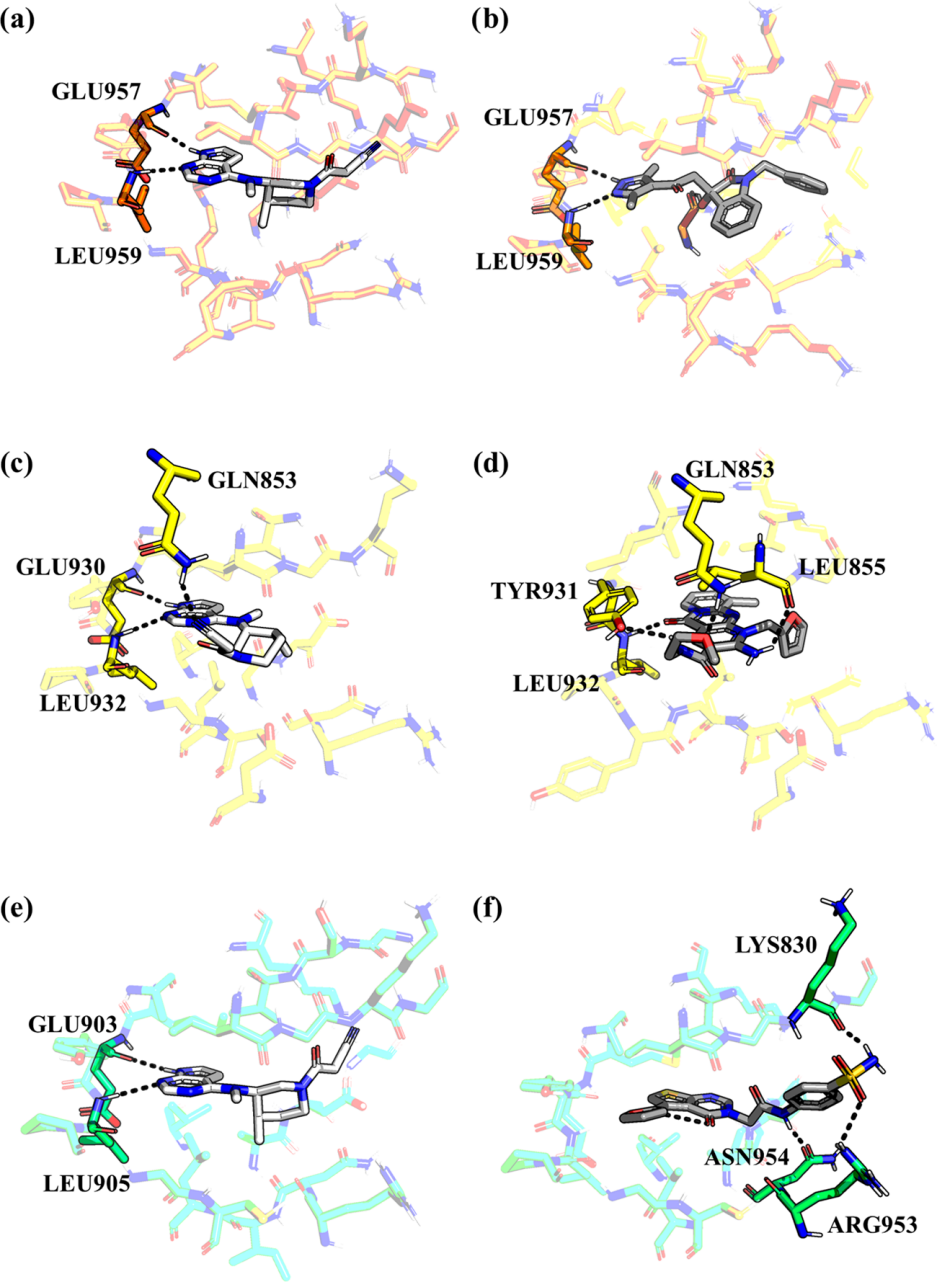
Ball and stick representation of the three top-ranked compounds for each JAK subtype: **5** bound to JAK1, **2** bound to JAK2, and **6** bound to JAK3 (panels b, d, and f, respectively). For comparison, the binding mode of the reference compound Tofacitinib is also reported (panels a, c, and e for JAK1, JAK2, and JAK3, respectively).

In Figure 9, we report the results of the docking calculations for the compounds showing the best value of the docking score for each JAK subtype together with the binding mode predicted for the reference compound (Tofacitinib). Interestingly, the pyrrolopyrimidine scaffold of Tofacitinib displays a consistent binding mode in all JAK subtypes, establishing hydrogen bonds with the backbone of residues in the hinge region (Glu957 and Leu959, Glu930 and Leu932, and Glu903 and Leu905 in JAK1, JAK2, and JAK3, respectively). Conversely, the cyanoacetyl-methyl piperidine substituent is differently oriented in the binding pocket of JAK2 compared to the other two subtypes. In JAK2, the specific orientation of the substituent allows the cyano group of Tofacitinib to establish an additional hydrogen bond with the side chain of Gln853, which is a residue not conserved in JAK1 and JAK3 (where the shorter asparagine and serine are found in the corresponding sequence position, respectively). As expected, hydrogen bonding with residues belonging to the hinge region of the enzyme is a shared feature among all selected compounds, even though the optimal geometry of the interaction is not always satisfied.

To refine the binding modes obtained through the docking procedure, we applied MD simulations to all complexes involving the prioritized molecules. By doing so, it is possible to unmask unreliable docking results by monitoring the stability of the binding mode over time. Accordingly, docking poses showing low stability, or even spontaneous dissociation events, during relatively short MD runs should be regarded as suspicious, probably the result of artifacts due to the several approximations introduced into the scoring function. Conversely, a meaningful docking pose will display stable and specific interactions with the target, showing a low root-mean-square deviation (RMSD) over time, with respect to the starting configuration. Even though more rigorous (but computationally much more demanding) approaches should be employed for unambiguously ruling out false positives from docking outcomes,^23^ a simple structural analysis of MD trajectories has proven to significantly improve the predictions of docking programs.^24,25^

**Figure 10:**
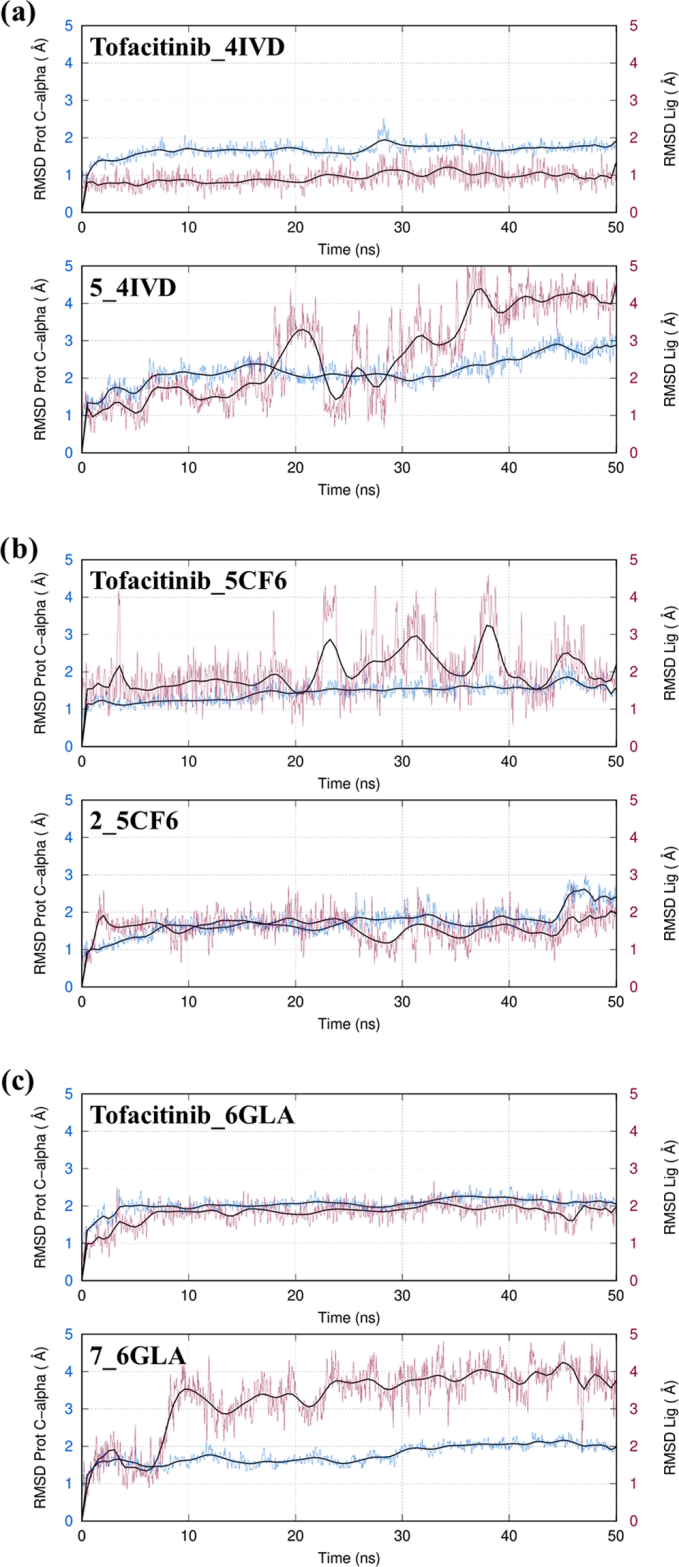
RMSD values of Cα atoms of the three JAK subtypes (a: JAK1; b: JAK2; and c: JAK3) in the complexes with the best-ranking molecule for each subtype and Tofacitinib along the 50 ns of MD simulations (blue lines). In purple, the RMSD computed using the heavy atoms of the ligands (after least-squares-fit superimposition to the Cα atoms of the protein) is also shown.

In Figure 10, we show that all investigated complexes reached a stable conformation during the time course of the simulations. In particular, Tofacitinib displayed a strikingly stable binding mode, especially in JAK1 and JAK3, whereas noticeable fluctuations were recorded when in complex with JAK2. The lower stability shown by Tofacitinib in JAK2 can be attributed to the different orientations of the cyanoacetyl-methyl piperidine substituent. High stability was observed for compound **2** bound to JAK2, while higher RMSD values were noticed for the binding modes of the other compounds. This structural stability was not observed for all investigated compounds. For example, in JAK1, two spontaneous dissociation events were observed (compounds **6** and **7**) even though stable binding modes were obtained in most of the cases. In particular, compounds **3** and **8** displayed remarkably stable interactions. In some cases (**1**, **2**, **4**, and **5**), large fluctuations of the ligand within the binding pocket were found, but the main interactions of docking pose were maintained. In general, greater stability and smaller fluctuations were observed in the case of JAK2, except for compound **6**, which changed the binding mode, and **4**, which left the binding site. Concerning JAK3, almost the entire pool of ligands showed low RMSD values, with the exception of compounds **1** and **8**, which left the binding pocket in the early stages of the simulations.

**Figure 11:**
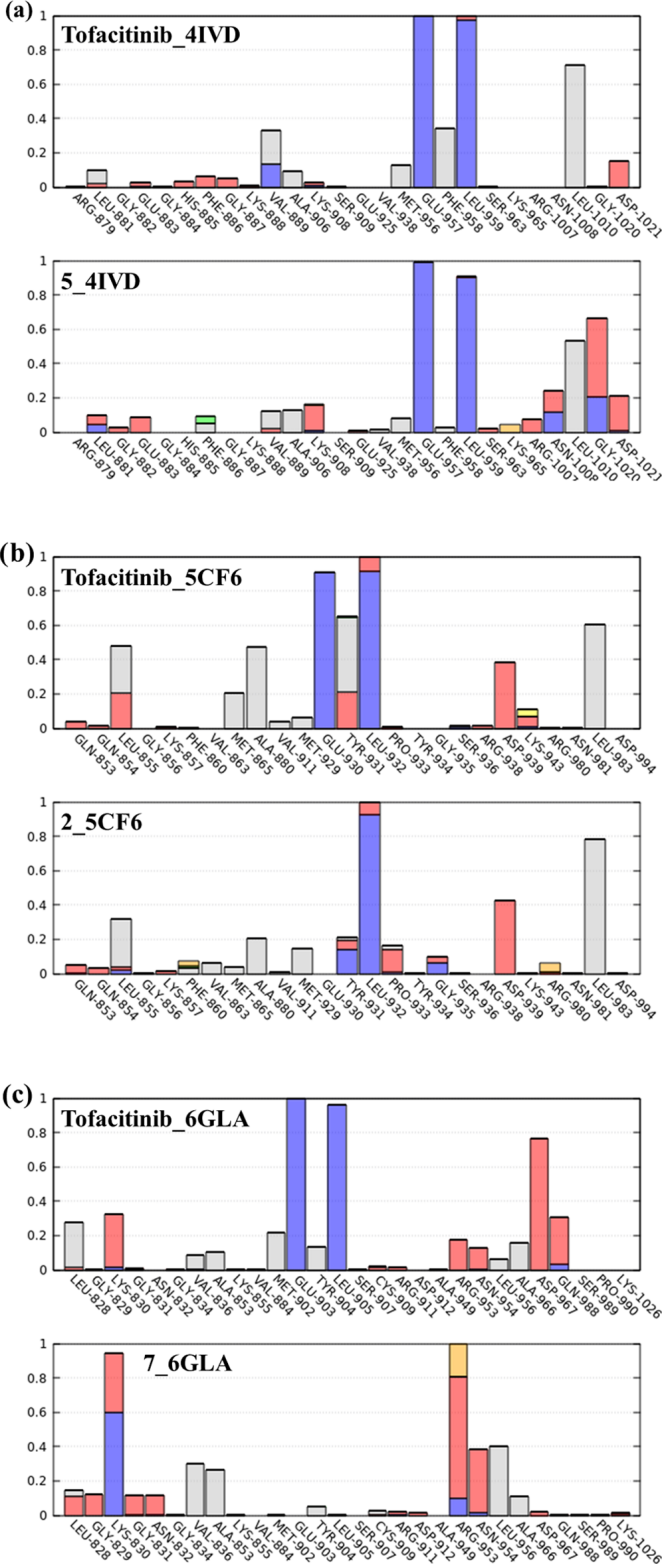
Protein–ligand contact interaction over the MD trajectory. Hydrogen bonds are shown in blue, water-mediated hydrogen bonds in red, hydrophobic interactions in gray, salt bridges in yellow, π–π interactions in green, and cation−π interactions in orange.

For the compounds that showed a stable binding mode, further characterizing the interactions preserved or gained during the MD simulations can be of some interest for grasping some aspects related to the potential selectivity toward specific JAK subtypes. In Figure 11, we show a per-residue interaction analysis of the persistence of the interaction for compounds **5**, **2**, and **6** bound to JAK1, JAK2, and JAK3, respectively. For comparison, the same plot obtained for Tofacitinib is also reported. From the plots, it can be inferred that for all JAK subtypes Tofacitinib preserved the key interactions with the residues belonging to the hinge region of the enzyme for the entire duration of the simulation (blue bars in Figure 11). These key interactions were also maintained in the simulations of the selected molecules, with the exception of compound **2**, which established a long-lasting hydrogen bond only with the backbone of Leu932 in JAK2. Interestingly, in JAK1 and JAK2, Tofacitinib was found to establish stable van der Waals interactions with a conserved leucine residue at the bottom of the binding site (Leu1010 and Leu983, respectively), while this interaction seems to be much less relevant in the case of JAK3 (Leu956). The same pattern for the same residue can be observed for compounds **5**, **2**, and **6** (bound to JAK1, JAK2, and JAK3, respectively). Regarding the other five prioritized molecules, we can summarize the following results. For JAK1, molecules like **1**, **3**, and **4** show an interaction pattern similar to the reference drug and the top scorer for the considered subtype (compound **5**). On the other hand, compounds that establish during more than 60% of the simulated period interactions with Asp1021 (**4**, **7**, and **8**) are found, with generally better docking score values. Therefore, this amino acid might contribute to the inhibitory activity against JAK1. Concerning JAK2, it is interesting to highlight how additional interaction not appreciated during the docking study with Leu983 is present in several compounds (Tofacitinib, **2**, and **6**) and seems to be key in obtaining favorable docking score values. Lastly, another interaction that was not appreciated in the docking results is represented by Leu905 in the case of JAK3. This interaction seems to be important for achieving good stability, especially if it is accompanied by interactions with Lys830 and/or Arg953 (**1**, **3**, and **7**), allowing us to establish more stable interactions with the protein (Table 7).

**Table 7:** Potential JAK Inhibitor Selectivity for Selected Compounds to be Tested *in vitro*: QSAR Model, Molecular Docking, and Dynamics Analysis

				docking score (kcal/mol)		
compound	predicted subtype			JAK1 (PDB-ID: 4IVD)	JAK2 (PDB-ID: 5CF6)	JAK3 (PDB-ID: 6GLA)
**1**	JAK1	JAK2		–7.06^b^	–7.61	–8.00
**2**	JAK1	JAK2		–6.82	–9.46	–6.96
**3**	JAK1	JAK2		–8.24	–7.31	–7.89
**4**	JAK1	JAK2		–6.81	–8.17	–7.26
**5**	JAK1^a^	JAK2		–9.05	–7.11	–6.90
**6**	JAK1^a^	JAK2	JAK3	–8.14	–9.03	–8.32
**7**	JAK1^a^		JAK3	–5.34	–7.34	–6.88
**8**	JAK1^a^	JAK2		–8.13	–6.28	–5.73

a Overlapping zone with other JAK inhibitor subtypes, nonabsolutely sure being correctly classified by this model.

b kcal/mol.

Analyzing the QSAR prediction, we see that four compounds are potentially JAK1 inhibitors, seven are inhibitors of JAK2, and only two would inhibit JAK3. When analyzing the data obtained in the molecular docking study, the criterion of counting how many compounds have obtained a docking score value of less than −7 kcal/mol for the different JAKs studied was applied. When looking at the table, it can be seen how five compounds obtain values lower than −7 kcal/mol for JAK1, seven for JAK2, and four for JAK3. Even though scoring functions are not enough sensitive for describing selectivity in general, we can observe an encouraging consensus between the predictions of the QSAR models and the indications returned by the docking scores. In particular, both strategies suggest a potential ability of the selected molecules to inhibit JAK1 and JAK2, with a slight preference over the latter subtype. This information was instrumental to direct the *in vitro* biological assays toward the JAK2 subtype.

### 2.4 Chemical Diversity of Potential JAK Inhibitors

The chemical diversity of potential JAK inhibitors (*in vitro* tested) compared to known JAK inhibitors has been analyzed. As reported in Section 4.4, all pairwise distances between molecules were computed from their fingerprints, using a metric derived from the Tanimoto coefficient for the corresponding binary strings.

Cluster analysis is depicted on a hierarchical clustering dendrogram panel (Figure S2) on supplementary material. In this dendrogram, analyzed data is divided into six clusters based on chemical diversity: cluster #1 (HBCH, hexabromocyclohexane), cluster #2 (**1)**, cluster #3 (**4**), cluster #4 (**8**), cluster #5 (45 molecules, known JAK inhibitors, and compounds **2**, **3**, **5**, and **6**), and cluster #6 (**7**).

The most populated cluster is cluster #5, where almost all known JAK inhibitors plus potential JAK inhibitors **2**, **3**, **5**, and **6** are found. Thus, there is a chemical similarity between these potential JAK inhibitors and already known ones. The rest of the potential JAK inhibitors (**1**, **4**, **7**, and **8**) belong to different clusters; therefore, a chemical diversity (potential hits with novel scaffolds) is pointed in relation to known JAK inhibitors.

### 2.5 *In Vitro* Tests

#### 2.5.1 MTT Cell Viability

**Figure 12:**
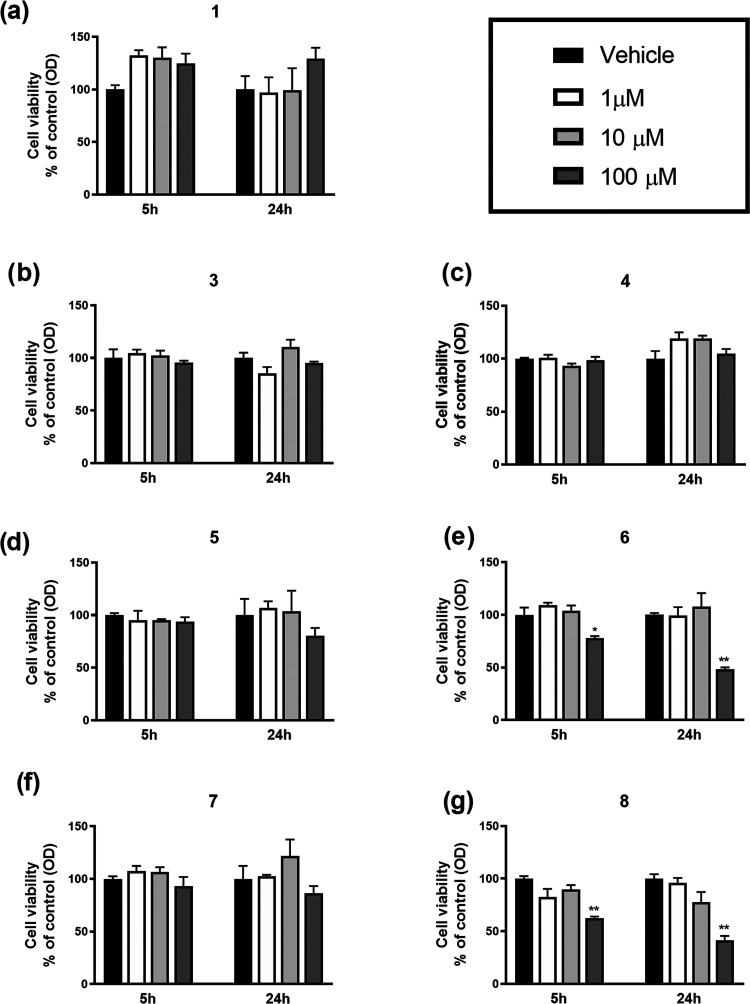
Cell viability of SH-SY5Y cells exposed to different concentrations of tested drugs for 5 and 24 h, evaluated by the MTT assay. Data are expressed as a percentage of OD values of treated cells compared to vehicle-treated ones and reported as the mean ± standard error of the mean (SEM) (*p < 0.05; **p < 0.01 vs the respective control, one-way analysis of variance (ANOVA) test followed by Dunnett’s test).

The MTT analysis revealed that the majority of JAK2 potential inhibitors did not evidence cytotoxic effects. In fact, SH-SY5Y cells exposed to the lower concentrations (1 and 10 μM) of the investigated substances did not show significant survival alterations after both 5 and 24 h (Figure 12).

Even though the majority of tested drugs did not cause changes in cell viability at 100 μM, a significant decrease was observed after the exposure to both **8** and **6** compounds, after either 5 or 24 h (*p < 0.05; **p < 0.01 vs the respective control) (Figure 12b,f). As a consequence, compounds **8** and **6** would not be utilized at higher concentrations and cautionally cannot be considered candidates for further study.

It is to be noted that **2** was not tested *in vitro* because of solubility problems even if it was one of the most promising candidates. It showed high specific JAK2 inhibition capability according to the docking analysis (Table 7).

Taken together, the obtained results suggest that the majority of selected compounds showed low cytotoxicity, thus stimulating further investigation to better define their possible utilization as candidates *in vivo*.

#### 2.5.2 JAK2 Activity Assay

The JAK2 inhibitory activity was tested for each compound from 0.1 nM to 100 μM. Tofacitinib, a well-known JAK pan inhibitor, was used as a positive control. All tested compounds exhibited a JAK2 inhibitory activity lower than Tofacitinib (Table 8). However, for compounds **4** and **7**, it has been possible to calculate the IC_50_ (half maximal inhibitory concentration) value in the adopted range of concentrations (Table 8).

**Table 8:** IC_50_ Values in the Enzymatic Assay for JAK2 Inhibition

compounds	IC_50_ (nM)
**1**	>10 000
**3**	>10 000
**4**	807 (643–1007)
**5**	>10 000
**6**	>10 000
**7**	637 (367–1076)
**8**	>10 000
**Tofacitinib**	31.4 (16–61)

a 95% confidence limits are shown in brackets.

**Figure 13:**
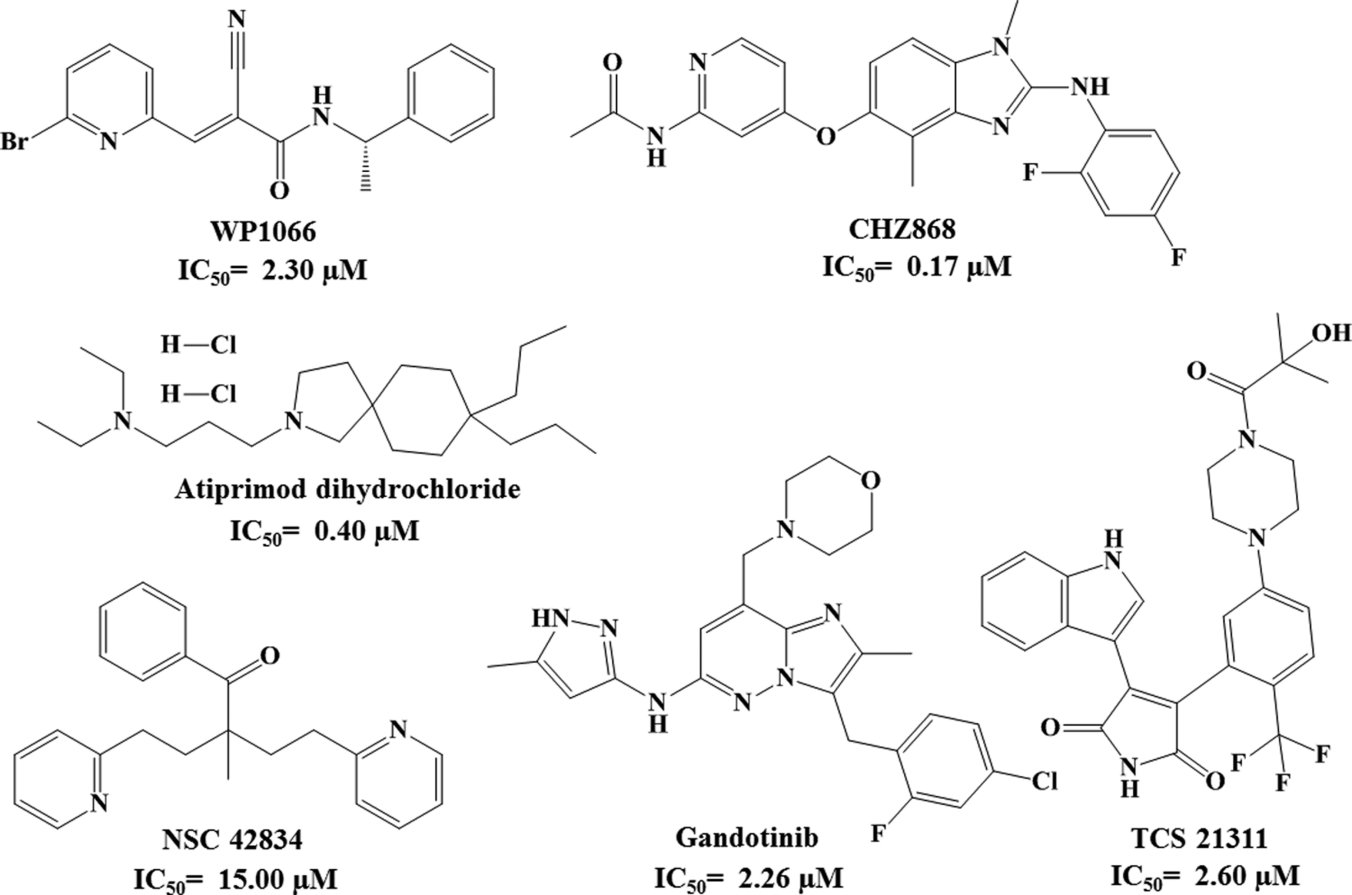
Chemical structure and IC_50_ (μM) for commercially available JAK2 inhibitors.^26−28^

As revealed by fingerprint-based cluster analysis, compounds **4** and **7** share no chemical similarity with already known JAK2 inhibitors; therefore, they can be considered as novel scaffolds for JAK2 inhibitors. Furthermore, compounds **4** and **7** show a JAK2 inhibitory activity comparable to that of commercially available JAK2 inhibitors (Figure 13).^26−28^

According to QSAR outcomes, all candidates were classified by DF_2_ as potential JAK2 inhibitors except for compound **7**. In contrast, molecular docking studies predicted for this molecule a higher level of affinity against JAK2 (see Table 7) compared with other JAK subtypes under study. In the particular case of **4** (second in JAK2 inhibition potency), there is a consensus between the QSAR model prediction and docking study, as it was predicted as a potential JAK2 inhibitor and its docking score for JAK2 was greater than the other JAK subtypes under analysis (Table 7).

**Figure 14:**
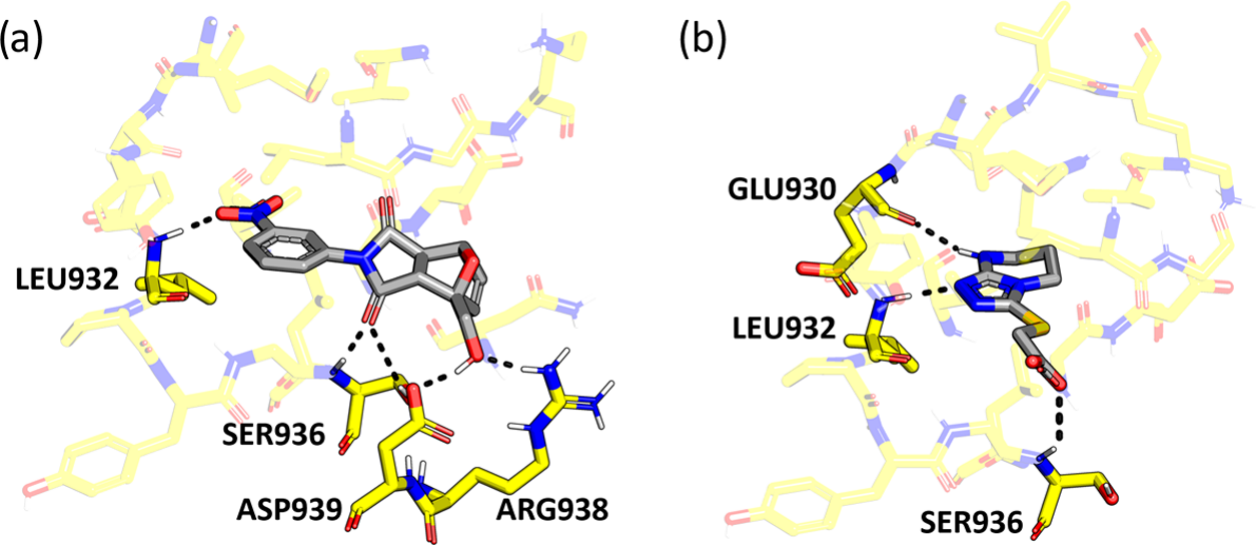
Amino acid interaction between *in vitro*-tested compounds **4** (a) and **7** (b) and JAK2 (PDB: 5CF6).

The analysis of which amino acid enzyme residues interact with compounds showing the highest *in vitro* inhibitory activity against JAK2 identifies Leu932 as the only residue in which both best compounds and the reference drug Tofacitinib coincide (see Figures 9 and 14). It can be deduced that the hydrogen-bond-type interactions established between compounds and the binding site of 5CF6 (JAK2) are essential for the activity. It can be hypothesized that in addition to Leu932, residue Glu930 (a residue with which it interacts Tofacitinib and **7**), Ser936, Asp939, and Arg938 might contribute to the inhibitory activity of the considered compounds against JAK2 (see Figures 9 and 14).

## 3 Conclusions

In this work, we have described a combined approach based on topological QSAR models, molecular docking, and molecular dynamics simulations for the discovery of novel JAK inhibitors that may be potentially useful to treat different autoimmune diseases and certain types of leukemia and, above all, for the control of the cytokine storm caused by SARS-CoV-2 infection. Among the eight prioritized compounds, two of them, **4** and **7**, showed a promising activity toward the inhibition of JAK2, with IC_50_ values of 0.81 and 0.64 μM, respectively, and displaying innovative scaffolds. The activity is comparable to that of other commercially available JAK2 inhibitors, and future lead optimization studies will be required for increasing the potency and tailoring the druglike properties of the selected molecules. In addition, the identified hit compounds could also be tested in cell-based assays and in animal models of disease related to the STAT/JAK pathway to characterize their efficacy and safety profiles.

## 4 Experimental: *In Silico* Modeling

### 4.1 QSAR Model

#### 4.1.1 Strategy to Identify Novel JAK Inhibitors

In this section, the strategy for identifying novel JAK inhibitors by applying QSAR based on LDA and MT is presented. As reported in Figure 1, the first step is creating a comprehensive database of compounds including both JAK inhibitors and structurally unrelated inactive compounds (or decoys). Once the database is prepared, topological descriptors for all data are calculated using AlvaDesc software.^29^

The data set is then divided into two groups, training and test sets. Considering the large data set of compounds, a significant percentage of the data is used as the test set (approximately 35%) during the construction of the general model. For the subtype-specific models, only the training set was used because of the short availability of compounds in the data set.

All of the models were built using the LDA method, considering only the training set. Then, they were validated through internal or external validation, and finally, the general model was used to perform a virtual screening of the SPECS Screening compounds library database (over 200 000 compounds available in 10 mg amounts or more) using the discriminant function DF_gen_. Conversely, the subtype-specific models were used after the screening for profiling the likelihood of the selected compounds to inhibit JAK1, JAK2, or JAK3 (through the usage of the discriminant functions DF_1_, DF_2_, and DF_3_, respectively).

#### 4.1.2 Data-Set Compilation

The database of JAK inhibitors was prepared using information from the literature^30,31^ and from commercial databases such as Tocris DB,^32^ ABCAM,^33^ and Selleck.^34^ In particular,

- Data set for the general model. Active compounds were JAK inhibitors retrieved from the literature^26,30,31^ and from the commercial database, and inactive compounds were taken from the Sigma-Aldrich catalog (therefore they act as putative inactive compounds, or decoys). A chemical similarity analysis was performed between active and inactive groups, by selecting compounds with similar MW, and the number of carbon, nitrogen, oxygen, and halogens atoms.
- Data set for the JAK1 subtype-specific model. Active compounds were commercial JAK1 inhibitors, and inactive compounds were commercial JAK2 and JAK3 inhibitors.
- Data set for the JAK2 subtype-specific model. Active compounds were commercial JAK2 inhibitors, and inactive compounds were commercial JAK1 and JAK3 inhibitors.
- Data set for the JAK3 subtype-specific model. Active compounds were commercial JAK3 inhibitors, and inactive compounds were commercial JAK1 and JAK2 inhibitors.

#### 4.1.3 Calculation of Descriptors

AlvaDesc software was employed for the calculation of the descriptors. Approximately 1500 indices, belonging to different categories like constitutional indices, ring descriptors, topological indices, walk and path counts, connectivity indices, information indices, 2D matrix-based descriptors, 2D autocorrelation, Burden eigenvalues, P_VSA-like descriptors, ETA indices, and edge adjacency indices, have been calculated.

#### 4.1.4 Statistical Analysis to Build the Model

To predict the JAK inhibitory activity, an LDA model was built (general model). A discriminant model can be defined as a classifier: it predicts a qualitative response from a specific observation, as for example, whether one molecule described by several topological indices shows a JAK inhibitor profile or not. An LDA model can be written as a discriminant function that is linear on the molecular indices
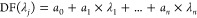
5where λ*_j_* are the topological indices characteristic of the molecule *j* and *a_i_* are the adjustable model parameters. The molecule *j* will be classified as a JAK inhibitor if DF is positive and as a non-JAK inhibitor if DF is negative.

As described above, one can calculate several topological descriptors for a specific molecule, but not all of them will actually be helpful to understand the property we are trying to model. Thus, it is necessary to choose a subset of indices from which the model will be built. We used the hybrid stepwise selection algorithm^35^ as implemented in STATISTICA.^36^ The algorithm proceeds iteratively. At every step, it may either add a variable to the model or remove a variable already present in the model, based on the *p*-value < or > 0.05. Once this procedure is terminated, the algorithm stops when the model reaches a maximum of 20 topological descriptors, which should be enough to minimize the bias of the model, but at the same time, still allowing an exhaustive search. The search was performed using as the selection criterion the minimization of Wilks’ λ. Wilks’ λ applied this way measures whether every parameter is contributing significantly to reducing the variance and not just the final classification results. The shorter the Wilks’ parameter value, the smaller the overlap of the active and inactive (λ = 0 would mean a perfect separation between the groups). Optimizing this statistic yields some confidence that the selected indices for a given model are statistically significant, hopefully allowing us to draw a trend valid for the whole set of molecules.

Finally, the Fisher–Snedecor *F* statistic parameter provides information related to the significance of each independent variable in explaining the dependent variable (in our particular case, JAK inhibition). The higher the *F* value, the more significant the variables are in a discriminant model.

#### 4.1.5 Pharmacological Distribution Diagram

A pharmacological distribution diagram (PDD) is a graphical representation that provides a direct way of visualizing the zones of minimum overlap between active and inactive compounds, as well as the region in which the probability of finding active compounds is the maximum.^37^ From a different perspective, a PDD is a frequency distribution diagram of dependent variables in which the ordinate represents the expectancy (probability of activity) and the abscissa represents the DF values in the range. For an arbitrary range of values of a given function, the expectancy of activity can be defined as *E*_a_ = *a*/(*i* + 1), where a is the number of active compounds in the range divided by the total number of active compounds and *i* is the number of inactive compounds in the interval divided by the total number of inactive compounds. The expectancy of inactivity is defined likewise as *E*_i_ = *i*/(*a* + 1). By means of these diagrams, it is easy to visualize the intervals in which there is a maximum probability to find new active compounds as well as the minimum probability to find inactive compounds.

#### 4.1.6 Validation of the Models

Two points of interest are (i) to estimate the correctness rate achievable with new molecules not previously used when training the model and (ii) to know the optimal model complexity, i.e., the most appropriate number of molecular indices to consider for making predictions without overfitting. Both cases are addressed by means of validation. The models employed in this paper were subjected to both internal and external validation.^38^

Once a predictive model is created, different validation approaches can be performed on two types of data: data used to build the model (internal validation) or data that was not used (external validation). The internal validation allows analyzing the robustness of the model, while the external validation allows understanding of the predictive performance of the model deteriorations when new data is provided.

For the general model, considering that the data set was large enough to leave aside randomly the 20% of the compounds to create a test set, an external validation was carried out. For the subtype-specific models, instead, the data set was not large enough to create a test set, so an internal validation was used with a 10-fold leave-some-out cross-validation (LSOCV). The procedure consists of leaving aside 20% of the training set as an artificial test set (7 compounds). The rest of the compounds, either active or inactive, are used for training the model, and the remaining one is used for cross-validation (Table S4). This is repeated 10 times, each time using a different group for cross-validation. At the end of LSOCV, the reliability of the discriminant function can be evaluated by a recalculation of the Wilks’ parameter (λ′) in each of the LSOCV cases. Similar values of λ and λ′ indicate a good validation of the model.

### 4.2 Molecular Docking Simulations

The crystal structures of JAK1, JAK2, and JAK3 were retrieved from Protein Data Bank (PDB-IDs: 4IVd, 5CF6, and 6GLA).^20−22^ The definition of the binding site pocket for the different JAK subtypes was provided by the coordinates of the cocrystallized ligands. Once the ligand and protein were prepared, the grid was generated according to the largest ligand and used as the reference to perform the cross-docking analysis (box size of 20 Å per side).

#### 4.2.1 Molecular Docking

The disordered regions of the proteins were reconstructed using the Prime-v34012 module of Schrodinger LLC (NY).^39^ Docking calculations were performed using the Schrödinger software suite molecular modeling package (version 2017-3),^39^ using default parameters unless otherwise reported. Known and potential JAK inhibitors were docked into different JAK subtype binding sites using Glide SP to evaluate their potential role against each JAK subtype.

#### 4.2.2 Cross-Docking Analysis

The Xglide tool from the Schrödinger software suite molecular modeling package (version 2017-3)^39^ was used to perform a cross-docking analysis for all of the selected cocrystallized JAK proteins and their ligands.

The docking accuracy was evaluated in terms of (i) the average RMSD values calculated between the positions of ligand atoms in the X-ray structure and the docked complex and (ii) the percentage of accurate poses having an RMSD < 2 Å (success rate).

### 4.3 Molecular Dynamics Simulations

MD simulations were performed using the Desmond code.^39^ All systems were solvated in an orthorhombic box (a margin of 10 Å between the solute and the side of the box was used in each dimension) with explicit TIP3P water molecules. All systems were neutralized, and an ionic salt concentration of 0.15 M of Na^+^ and Cl^–^ was added. Atomistic interactions were calculated with the OPLS3e force field (Desmond 5.9). After the construction of the solvent environment, each complex system was composed of about 35 000 atoms. Before equilibration and the long-production MD simulations, the systems were minimized and pre-equilibrated using the default relaxation routine implemented in Desmond. A multiple time-stepping of 2, 2, and 6 fs was used. The system equilibration was done via NVT and NPT ensembles using the SHAKE algorithm and by bringing the temperature up to 300 K and pressure up to 1 bar. Then, the systems were submitted in 10 and 50 ns MD simulations for equilibration and production MD runs for each system. Finally, 50 ns nonconstrained MD simulation was performed for each system, and the coordinates were saved for every 5 ps.

### 4.4 Chemical Diversity of Potential JAK Inhibitors

To determine the chemical diversity between the known and the identified potential JAK inhibitors (*in vitro* tested), the Tanimoto coefficient was employed. In particular, pairwise fingerprinting, generated using default atom-typing scheme Carthart atom types (Car), was calculated for all known and potential JAK inhibitors using the Canvas application of the Schrodinger Suite.^39^ Each compound is mapped to a binary string (32 bits long using default Canvas settings), which serves as a compact one-dimensional descriptor of the chemical structure. Finally, a similarity matrix, based on the Tanimoto similarities between each set of fingerprints, was calculated. The resulting distance matrix was used to generate a hierarchical clustering of the compounds, and by application of an appropriate distance threshold, the hierarchical grouping gave rise to a set of defined clusters with a specific compound membership. Chemical similarities between analyzed molecules (known and potential JAK inhibitors) were represented by dendritic hierarchal clustering performed using the Kelley criterion.

## 5 Experimental: *In Vitro* Assays

### 5.1 Cell Culture

Human SH-SY5Y neuroblastoma cells purchased from ICLC-IST (Genoa, Italy), were cultured in Dulbecco’s modified Eagle’s medium (DMEM), supplemented with 10% (v/v) fetal bovine serum (FBS), 100 units/mL penicillin, 100 μg/mL streptomycin, and 2 mM glutamine. Cells were incubated at 37 °C in a humidified atmosphere containing 5% CO_2_ and were allowed to reach 80% confluence before starting treatments. All reagents employed for cell culture were purchased from Lonza (Milan, Italy).

### 5.2 Cell Viability Assay

Drugs effects on cell viability were measured using the MTT [3-(4,5-dimethylthiazol-2-yl)-2,5-diphenyltetrazolium bromide] assay.^40^ All reagents were purchased from Sigma-Aldrich (Milan, Italy) unless otherwise indicated. Briefly, cells were plated on 24-well plates at a density of 3 × 10^4^ cells/well and were grown to reach 80% confluence. Cells were treated with vehicle (DMEM containing 0.1% dimethyl sulfoxide (DMSO)) or drugs dissolved in vehicle at the final concentrations of 1, 10, or 100 μM. After 5 or 24 h, the culture medium was removed and replaced with fresh DMEM containing the MTT solution (0.5 mg/mL) and cells were incubated in the dark for 3 h at 37 °C. After supernatant removal, a dimethyl sulfoxide/ethanol (4:1) mixture was added to each well to dissolve formazan crystals. The optical densities (ODs) were then recorded using a microplate spectrophotometer (GENios Tecan, Austria) at 590 nm. Results were expressed as a percentage of OD values of drug-treated cell cultures compared to vehicle-treated ones.

### 5.3 JAK2 Assay

The ability of tested compounds to inhibit JAK2 activity was performed using the JAK2 assay kit (BPS Bioscience, San Diego, CA). According to the manufacturer’s instructions, the assay was performed in a white 96-well plate, in a total volume of 50 μL, adding a 5× kinase assay buffer, ATP (500 μM), protein tyrosine kinase substrate (10 mg/mL), water, tested molecules diluted in 0.1% DMSO solution, and JAK2 enzyme (2.5 ng/μL).

The plate was incubated for 45 min at 30 °C, and then, the protein kinase activity was revealed adding Kinase Glo Max (Promega). After 15 min of incubation at room temperature, the luminescence was measured by EnSpire Multimode Plate Reader (Perkin Elmer). All measurements were performed in triplicates for each compound, and the concentration range tested was 0.1–10 000 nM.

### 5.4 Statistical Analysis

MTT assay results were statistically analyzed by one-way ANOVA followed by Dunnett’s test and are reported as the mean of values ± SEM. Statistical analysis was performed using GraphPad Prism software (version 8.00 for Windows, GraphPadSoftware, San Diego CA), and statistical significance was set at *p* < 0.05.

## Supporting Information Available

Descriptor values, classification of compounds and probability of activity for all data sets of models 1–4 (Tables S1–S5); leave-some-out validation test for DF_2-4_ (Tables S6–S8); docking score values from potential JAK inhibitors selected by molecular topology (Table S9); cross-docking analysis for JAK1, JAK2, and JAK3 subtypes (Figure S1); similarity-cluster analysis dendrogram (Figure S2) (PDF)
