# Supplementary material for: Targeting the JAK/STAT Pathway: A Combined Ligand- and Target-Based Approach
Source: J Chem Inf Model. 2021 May 17;61(6):3091–108. doi: 10.1021/acs.jcim.0c01468 (PMC8491162; doi:10.1021/acs.jcim.0c01468)
Supplement: Supplementary file 1 — ci0c01468_si_001.pdf [file ci0c01468_si_001.pdf]

Supporting Information

Targeting the JAK/STAT Pathway: a Combined Ligand- and Target-Based Approach

Maria Galvez-Llompart,[a,b]* Riccardo Ocello,[c] Laura Rullo,[c] Serena Stamatakos, [c] Irene Alessandrini,[c] Riccardo Zanni,[a] Iñaki Tuñón,[a] Andrea Cavalli,[c,d] Sanzio Candeletti,[c] Matteo Masetti,[c] Patrizia Romualdi,[c#] Maurizio Recanatini.[c#]

[a]Department of Physical Chemistry, University of Valencia, Av. Vicente Estelles s/n, 46100 Burjassot (Valencia), Spain.
[b]Instituto de Tecnología Química (UPV- CSIC) Universidad Politécnica de Valencia Av. Naranjos s/n,46022 Valencia, Spain
[c]Department of Pharmacy and Biotechnology, Alma Mater Studiorum-University of Bologna, via Belmeloro 6, 40126 Bologna, Italy
[d] Italian Institute of Technology (IIT), Via Morego 30, 16163, Genoa, Italy
[#]Co-last author
*Corresponding autor. Maria Galvez-Llompart, Av. Vicente Estelles s/n, 46100 Burjassot (Valencia), Spain.
E-mail address: maria.galvez@uv.es (M. Galvez-Llompart)

Table of Contents

1. Tables indexed in manuscript…………………………………………………………..S3

1.1. Table S1. Value of descriptors from training set compounds, classification as JAK inhibitors by DF_1 and probability of activity……………………………………………….S3

1.2. Table S2. Value of descriptors from external test set compounds, classification as JAK inhibitors by DF_1 and probability of activity……………………………………………….S6

1.3. Table S3. Value of descriptors from all training set compounds, classification as JAK inhibitors by DF_2 and probability of activity……………………………………………….S8

1.4. Table S4. Value of descriptors from all training set compounds, classification as JAK inhibitors by DF_3 and probability of activity………………………………………………..S9

1.5. Table S5. Value of descriptors from all training set compounds, classification as JAK inhibitors by DF_4 and probability of activity………………………………………………S10

1.6. Table S6. Leave-some-out validation test for DF_2 by applying the criteria of leaving the 25% of the data set out as test set……………………………………………………………..S11

1.7. Table S7. Leave-some-out validation test for DF_3 by applying the criteria of leaving the 25% of the data set out as test set……………………………………………………………..S12

1.8. Table S8. Leave-some-out validation test for DF_4 by applying the criteria of leaving the 25% of the data set out as test set……………………………………………………………..S13

1.9. Table S9. Docking score values from potential JAK inhibitors selected by Molecular Topology……………………………………………………………………………………..S14

2. Cross-docking analysis: results……………………………………………………..S16

3. Similarity-cluster analysis: Fig S2......……………………………………………..S17

1. Tables indexed in manuscript

1.1. Table S1. Value of descriptors from all training set compounds, classification as JAK inhibitors by DF_{gen} and probability of activity.

Compound	SRW05	CIC2	GATS6m	DF_1	Class.	P.A.
**Active group**						
TCJL37	3.932	0.324	0.724	4.009	A	0.982
Cerdulatinib	3.714	1.116	0.73	2.933	A	0.949
Baricitinib	3.045	0.529	0.831	2.707	A	0.937
Filgotinib	3.932	1.088	0.938	2.426	A	0.919
TCS21311	3.045	1.300	0.75	2.125	A	0.893
Decernotinib	2.398	0.745	0.791	2.09	A	0.89
Itacitinib	3.045	0.849	0.941	1.974	A	0.878
Gandotinib	3.045	0.916	0.932	1.932	A	0.873
Lestaurtinib	3.714	1.014	1.091	1.809	A	0.859
Nifuroxazide	2.398	0.742	0.891	1.75	A	0.852
AZD1480	2.398	0.481	0.992	1.694	A	0.845
Cercosporamide	2.398	0.548	0.973	1.684	A	0.843
Tofacitinib	2.398	0.5	1.011	1.609	A	0.833
PF 06551600malonate	2.398	0.519	1.03	1.521	A	0.821
Solcitinib	3.932	1.253	1.162	1.473	A	0.814
Peficitinib	2.398	1.059	0.876	1.447	A	0.81
AC-430	2.398	0.935	0.932	1.393	A	0.801
Go6976	3.434	1.122	1.135	1.315	A	0.788
CEP33779	2.398	1.381	0.811	1.312	A	0.788
Oclacitinib	2.398	1.033	0.938	1.266	A	0.78
FM-381	3.434	1.147	1.159	1.205	A	0.769
Curcumol	3.045	1.404	0.986	1.204	A	0.769
N-(3-(1-(2-(methylsulfonyl)ethyl)piperidin-4-yl)phenyl)-8-(4-(methylsulfonyl)phenyl)-[1,2,4]triazolo[1,5-a]pyridin-2-amine	2.398	1.29	0.944	0.96	A	0.723
Pyridone6	2.398	0.975	1.049	0.951	A	0.721
WHI-P97	0	0.96	0.524	0.856	A	0.702
Hexabromocyclohexane	0	2.585	0	0.841	A	0.699
Pacritinib	2.398	1.306	0.980	0.817	A	0.694
Fedratinib	2.398	1.429	0.958	0.756	A	0.68
XL019	2.398	1.402	0.980	0.71	A	0.67
MS-1020	2.398	0.984	1.119	0.699	A	0.668
WP1066	0	1.077	0.580	0.534	A	0.63
AG490	3.045	0.903	1.346	0.528	A	0.629
CucurbitacinI	2.398	1.827	0.979	0.241	A	0.56
Curvularin	0	0.994	0.812	-0.169	I	0.458
R-348	0	0.82	1.001	-0.623	I	0.349

Atiprimod	2.398	2.982	0.869	-0.665	I	0.34
Momelotinib	0	1.255	0.905	-0.778	I	0.315
ZM39923hydrochloride	0	1.89	0.706	-0.803	I	0.309
ZM449829	0	1.267	0.926	-0.862	I	0.297
TG101209	0	1.583	0.945	-1.28	I	0.218
FLLL32	0	2.22	1.019	-2.243	I	0.096
NSC33994	0	2.332	1.056	-2.493	I	0.076
**Inactive group**						
118337-09-0	0	3.63	1.866	-6.712	I	0.001
10273-86-6	0	1.752	1.276	-2.601	I	0.069
100-93-6	0	1.932	1.199	-2.539	I	0.073
101086-83-3	0	1.798	1.234	-2.51	I	0.075
10128-55-9	0	1.806	1.229	-2.5	I	0.076
108045-24-5	0	2.917	0.767	-2.155	I	0.104
103348-49-8	0	2.363	0.945	-2.148	I	0.105
113446-02-9	0	1.602	1.177	-2.096	I	0.109
109562-39-2	0	1.66	1.133	-2.009	I	0.118
102-05-6	0	2.227	0.940	-1.979	I	0.121
122020-32-0	0	1.671	1.119	-1.975	I	0.122
114145-29-8	2.398	0.766	1.941	-1.871	I	0.133
102023-64-3	0	1.972	0.972	-1.806	I	0.141
107199-98-4	0	1.397	1.112	-1.644	I	0.162
113446-04-1	0	1.548	1.062	-1.64	I	0.162
10505-82-5	0	0.815	1.298	-1.632	I	0.163
S728241	0	1.084	1.210	-1.632	I	0.164
101430-18-6	0	1.412	1.101	-1.623	I	0.165
S685933	0	1.391	1.087	-1.553	I	0.175
109244-58-8	0	1.471	1.034	-1.459	I	0.189
124131-78-8	0	1.935	0.878	-1.442	I	0.191
S736805	0	1.086	1.152	-1.435	I	0.192
103579-16-4	2.398	3.312	0.977	-1.405	I	0.197
S727776	0	1.229	1.093	-1.393	I	0.199
104881-72-3	0	1.272	1.078	-1.388	I	0.2
119483-25-9	0	2.219	0.744	-1.299	I	0.214
108321-41-1	0	1.411	1.005	-1.294	I	0.215
109-20-6	0	1.635	0.923	-1.264	I	0.22
1237161-19-1	0	2.223	0.719	-1.219	I	0.228
119-15-3	0	1.367	0.981	-1.165	I	0.238
S231193	0	0.843	1.124	-1.07	I	0.255
124245-33-6	3.045	0.63	1.892	-1.039	I	0.261
102185-38-6	0	1.273	0.967	-1.012	I	0.267
124110-19-6	0	2.098	0.688	-0.975	I	0.274
114163-70-1	0	1.661	0.789	-0.833	I	0.303
117153-55-6	0	0.816	1.052	-0.793	I	0.312

100929-99-5	0	1.499	0.806	-0.712	I	0.329
S480525	0	0.969	0.952	-0.622	I	0.349
108124-93-2	0	1.554	0.731	-0.514	I	0.374
119-24-4	0	0.457	1.074	-0.469	I	0.385
105393-87-1	2.398	1.481	1.294	-0.452	I	0.389
S469734	0	1.238	0.808	-0.428	I	0.395
106917-30-0	2.398	2.95	0.751	-0.227	I	0.444
115408-94-1	0	0.995	0.796	-0.116	I	0.471
103192-42-3	0	1.067	0.766	-0.093	I	0.477
124809-76-3	2.398	1.875	0.990	0.148	A	0.537
123334-10-1	0	1.263	0.614	0.21	A	0.552
124110-18-5	3.045	1.956	1.087	0.242	A	0.56
109083-74-1	2.398	1.4	1.085	0.353	A	0.587
1251-85-0	3.045	2.033	0.999	0.46	A	0.613
120340-91-2	3.045	1.672	1.104	0.5	A	0.622
1016-05-3	2.398	1.826	0.88	0.58	A	0.641
1156-32-7	2.398	1.062	1.076	0.761	A	0.681
121593-77-9	2.398	1.367	0.902	1.018	A	0.735
112866-98-5	2.398	1.635	0.812	1.026	A	0.736
61773-91-9	2.398	0.829	1.056	1.086	A	0.748
105098-97-3	2.398	1.081	0.821	1.614	A	0.834
105379-24-6	3.434	1.86	0.696	2	A	0.881
1063-77-0	4.263	1.326	1.016	2.158	A	0.896

P.A.: probability of activity

1.2. Table S2 . Value of descriptors from all external test set compounds, classification as JAK inhibitors by DF_{gen} and probability of activity.

Compound	SRW05	CIC2	GATS6m	DF_1	Class.	P.A.
**Active group**						
1-(2-cyano-1-cyclopropylethyl)-3-((4-(methylsulfinyl)phenyl)amino)-1H-pyrazole-4-carboxamide	3.932	0.699	0.868	3.1	A	0.957
AT9283	4.111	0.721	0.905	3.091	A	0.957
BMS-911543	4.71	1.084	0.961	2.972	A	0.951
1-(2-cyano-1-cyclopropylethyl)-3-((4-(N,N-dimethylsulfamoyl)phenyl)amino)-1H-pyrazole-4-carboxamide	3.932	0.91	0.851	2.92	A	0.949
ABT-494	3.434	0.674	0.867	2.731	A	0.939
N-(5-(4-((1,1-dioxidothiomorpholino)methyl)phenyl)-5,8-dihydro-[1,2,4]triazolo[1,5-a]pyridin-2-yl)cyclopropanecarboxamide	3.932	0.982	0.937	2.548	A	0.927
N-(5-(4-((1,1-dioxidothiomorpholino)methyl)phenyl)-[1,2,4]triazolo[1,5-a]pyridin-2-yl)cyclopropanecarboxamide	3.932	1.088	0.938	2.426	A	0.919
(S)-2-((S)-2,4-dimethylpiperazin-1-yl)-N-(3-(2-((3-ethoxy-1-methyl-1H-pyrazol-4-yl)amino)-5-fluoropyrimidin-4-yl)-1H-indol-7-yl)propanamide	3.045	1.004	0.886	1.99	A	0.88
(R)-2-((S)-3,4-dimethylpiperazin-1-yl)-N-(3-(2-((3-methoxy-1-methyl-1H-pyrazol-4-yl)amino)-5-methylpyrimidin-4-yl)-1H-indol-7-yl)propanamide	3.045	0.928	0.915	1.976	A	0.878
PF-04965842	2.398	0.768	0.82	1.965	A	0.877
Ruxolitinib	3.434	0.78	1.069	1.922	A	0.872
PF-06651600	2.398	0.603	0.948	1.708	A	0.847
AZ-960	2.398	0.739	0.907	1.7	A	0.846
3-(azepan-1-yl)-1-(naphthalen-2-yl)propan-1-one	2.398	0.859	0.885	1.641	A	0.838
3-((3R,4R)-4-methyl-3-(methyl(7H-pyrrolo[2,3-d]pyrimidin-4-yl)amino)piperidin-1-yl)-3-oxopropanenitrile	2.398	0.512	1.003	1.623	A	0.835
(R)-3-methoxy-N-(3-(2-((3-methoxy-1-methyl-1H-pyrazol-4-yl)amino)-5-methylpyrimidin-4-yl)-1H-indol-7-yl)-2-(4-methylpiperazin-1-yl)propanamide	3.045	1.076	1.048	1.356	A	0.795
(S)-2-(dimethylamino)ethyl 4-(1-(4-carbamoyl-3-((4-fluorophenyl)amino)-1H-pyrazol-1-yl)-2-cyanoethyl)piperidine-1-carboxylate	2.398	0.985	0.987	1.153	A	0.76
1-(tert-butyl)-3-(4-chlorophenyl)-1H-pyrazolo[3,4-d]pyrimidin-4-amine	2.398	1.278	0.898	1.128	A	0.755
WHI-P154	0	0.895	0.563	0.792	A	0.688
(S)-tert-butyl 4-(1-(4-carbamoyl-3-((4-fluorophenyl)amino)-1H-pyrazol-1-yl)-	2.398	1.117	1.096	0.629	A	0.652

2-cyanoethyl)piperidine-1-carboxylate						
3-((4-(N-benzylsulfamoyl)phenyl)amino)-1-((1S,2S)-2-cyanocyclohexyl)-1H-pyrazole-4-carboxamide	2.398	1.188	1.075	0.624	A	0.651
NVP-BSK805	2.398	1.088	1.137	0.525	A	0.628
1-methyl-3-(3-(quinoxalin-2-yl)phenyl)thiourea	0	1.072	1.004	-0.913	A	0.286
JANEX-1	0	1.109	1.046	-1.097	I	0.25
WHI-P131	0	1.109	1.046	-1.097	I	0.25
3-(azepan-1-yl)-1-(naphthalen-2-yl)propan-1-one	0	1.725	0.966	-1.512	I	0.181
**Inactive group**						
103-19-5	0	2.117	1.882	-5.082	I	0.006
10329-75-6	0	2.252	1.326	-3.328	I	0.035
100527-04-6	0	1.517	1.524	-3.191	I	0.04
10536-58-0	0	3.255	0.896	-2.971	I	0.049
102751-76-8	0	2.482	1.127	-2.902	I	0.052
1064-10-4	0	4.159	0.364	-2.156	I	0.104
111-21-7	0	2.31	0.952	-2.111	I	0.108
118233-09-3	0	1.099	1.32	-2.026	I	0.117
122122-25-2	0	2.198	0.923	-1.889	I	0.131
108474-20-0	0	1.475	1.144	-1.84	I	0.137
115841-75-3	0	1.646	0.967	-1.428	I	0.193
121894	0	2.065	0.828	-1.415	I	0.195
101-85-9	0	1.218	1.069	-1.298	I	0.215
1214-75-1	0	1.152	1.047	-1.15	I	0.241
1235651-69-0	0	2.115	0.733	-1.145	I	0.241
1026-05-7	0	1.49	0.927	-1.117	I	0.247
114329-10-1	0	2.252	0.665	-1.066	I	0.256
121-79-9	0	0.899	1.087	-1.004	I	0.268
100381-45-1	0	1.419	0.774	-0.511	I	0.375
1098-60-8	0	1.576	0.633	-0.203	I	0.449
1175-06-0	2.398	2.502	0.879	-0.169	I	0.458
123359-43-3	2.398	1.724	1.057	0.09	I	0.523
1154-39-8	0	2.405	0.209	0.327	I	0.581
10184-96-0	2.398	1.145	1.087	0.63	A	0.653
54-42-2	2.398	0.286	1.285	0.909	A	0.713
115-27-5	3.434	0.9	1.315	0.948	A	0.721
118-36-5	2.398	0.94	1.034	1.04	A	0.739
1138-15-4	2.398	0.815	0.963	1.423	A	0.806
652-37-9	2.398	0.723	0.989	1.437	A	0.808
R171247	2.398	0.932	0.882	1.569	A	0.828
7029-96-1	2.398	0.796	0.89	1.694	A	0.845
2140-65-0	3.045	0.563	1.093	1.772	A	0.855

1.3. Table S3. Value of descriptors from all training set compounds, classification as JAK inhibitors by DF_1 and probability of activity.

Compound	PCD	MATS5i	DF	Class.	P.A.
**Active group**					
Baricitinib	3.18	-0.248	7.254	A	0.999
Ruxolitinib	3.068	-0.184	5.519	A	0.996
PF-04965842	2.15	-0.056	3.356	A	0.966
Oclacitinib	2.121	-0.052	3.289	A	0.964
Itacitinib	3.98	-0.16	2.991	A	0.952
Solcitinib	3.36	-0.12	2.966	A	0.951
Tofacitinib	2.176	0.008	1.323	A	0.79
Cerdulatinib	2.464	0.012	0.633	A	0.653
Filgotinib	3.478	-0.019	-0.364	I	0.41
Momelotinib	3.617	-0.02	-0.6	I	0.354
ZM39923hydrochloride	3.031	0.03	-1.005	I	0.268
**Inactive group**					
AT9283	3.111	0.171	-5.489	I	0.004
TG101209	3.554	0.127	-5.007	I	0.007
JANEX-1	3.374	0.1	-3.842	I	0.021
WHI-P154	3.399	0.098	-3.812	I	0.022
WHI-P97	3.418	0.095	-3.746	I	0.023
NVP-BSK805	4.392	0.024	-3.453	I	0.031
Gandotinib	3.593	0.064	-3.132	I	0.042
AZD1480	2.859	0.097	-2.75	I	0.06
Go6976	5.507	-0.071	-2.686	I	0.064
CEP33779	4.531	-0.021	-2.352	I	0.087
WP1066	2.488	0.087	-1.702	I	0.154
Hexabromocyclohexane	0	0.233	-1.406	I	0.197
Pacritinib	4.049	-0.021	-1.401	I	0.198
Decernotinib	3.253	0.028	-1.376	I	0.202
XL019	3.5	-0.007	-0.766	I	0.317
FLLL32	2.887	0.018	-0.36	I	0.411
PF 06551600malonate	2.155	0.036	0.503	A	0.623
BMS-911543	3.593	-0.08	1.3	A	0.786

1.4. Table S4. Value of descriptors from all training set compounds, classification as JAK inhibitors by DF_2 and probability of activity.

Compound	GATS4e	JGI8	Eig05_AEA(dm)	DF	CLASS	P.A.
**Active group**						
Cerdulatinib	1.264	0.008	2.769	5.616	A	0.996
Baricitinib	1.456	0.011	2.555	4.734	A	0.991
Pacritinib	1.085	0.006	2.598	4.432	A	0.988
Hexabromocyclohexane	1.145	0	1.833	4.357	A	0.987
Momelotinib	1.007	0.006	2.701	4.143	A	0.984
Filgotinib	1.121	0.009	2.632	3.253	A	0.963
XL019	0.987	0.006	2.505	2.921	A	0.949
TG101209	1.063	0.011	2.753	2.491	A	0.923
Go6976	0.99	0.009	2.69	2.275	A	0.907
Gandotinib	0.9	0.009	2.745	2.125	A	0.893
BMS-911543	1.246	0.015	2.775	2.014	A	0.882
FLLL32	1.153	0.011	2.442	1.866	A	0.866
Tofacitinib	1.012	0.008	2.381	1.832	A	0.862
WP1066	1.097	0.009	2.334	1.696	A	0.845
AT9283	0.747	0.006	2.663	1.533	A	0.822
Ruxolitinib	0.927	0.006	2.156	0.624	A	0.651
CEP33779	0.841	0.01	2.675	0.466	A	0.614
AZ-960	0.917	0.013	2.794	0.238	A	0.559
NVP-BSK805	0.799	0.011	2.664	-0.235	I	0.442
PF-04965842	1.213	0.014	1.759	-2.721	I	0.062
**Inactive group**						
Peficitinib	0.74	0.021	2.287	-8.126	I	0
ZM39923 hydrochloride	0.842	0.01	1.917	-2.979	I	0.048
Oclacitinib	1.078	0.013	1.919	-2.548	I	0.073
WHI-P154	0.973	0.013	2.221	-2.141	I	0.105
WHI-P97	0.933	0.014	2.437	-1.893	I	0.131
JANEX-1	0.968	0.012	2.219	-1.587	I	0.17
AZD1480	0.874	0.012	2.395	-1.498	I	0.183
PF 06551600 malonate	1.233	0.013	1.941	-0.948	I	0.279
Decernotinib	0.944	0.013	2.533	-0.745	I	0.322
Itacitinib	0.652	0.011	3.138	0.639	A	0.654

1.5. Table S5. Value of descriptors from all training set compounds, classification as JAK inhibitors by DF_3 and probability of activity.

Compounds	GATS8m	GATS5e	GATS5i	P_VSA_LogP_5	DF_4	Class.	P.A.
**Active group**							
AZD1480	1.87	0.709	0.788	76.766	7.253	A	0.999
Filgotinib	1.023	0.584	1.017	10.659	4.199	A	0.985
Decernotinib	1.158	0.522	0.707	87.847	3.663	A	0.975
WHI-P97	1.618	0.915	0.895	72.7	3.013	A	0.953
WHI-P154	1.518	0.88	0.884	69.645	2.935	A	0.949
ZM39923 hydrochloride	0.675	0.628	0.943	14.641	2.267	A	0.906
AT9283	1.135	1.183	0.821	47.416	1.232	A	0.774
PF 06551600 malonate	0.867	1.141	0.877	27.977	0.4	A	0.598
JANEX-1	1.073	0.849	0.876	66.59	0.38	A	0.594
Cerdulatinib	0.902	0.941	0.935	40.4	-0.061	I	0.484
Tofacitinib	0.952	0.811	0.904	62.161	-0.298	I	0.426
**Inactive group**							
Baricitinib	0.888	0.558	1.229	77.321	-5.311	I	0.005
FLLL32	0.887	1.298	0.963	71.344	-5.308	I	0.005
Itacitinib	1.252	0.727	0.927	148.347	-5.242	I	0.005
PF-04965842	0.824	1.483	0.978	31.101	-3.714	I	0.024
Pacritinib	1.028	1.144	1.02	61.496	-3.419	I	0.032
TG101209	1.134	1.678	0.824	64.324	-3.205	I	0.039
BMS-911543	1.172	1.498	1.114	28.303	-3.151	I	0.041
Hexabromocyclohexane	0	1.311	0.755	0	-2.544	I	0.073
XL019	0.837	1.07	0.983	44.395	-2.34	I	0.088
Momelotinib	0.954	0.718	0.967	75.883	-1.797	I	0.142
Go6976	1.028	0.846	1.076	51.521	-1.624	I	0.164
NVP-BSK805	0.978	0.939	0.957	61.025	-1.561	I	0.173
WP1066	0.866	1.149	0.877	40.187	-0.675	I	0.337
CEP33779	0.931	0.949	0.969	38.859	-0.279	I	0.431
Gandotinib	0.889	0.723	0.909	60.712	-0.159	I	0.46
Oclacitinib	0.955	1.045	0.976	31.101	-0.133	I	0.467
AZ-960	1.202	0.637	0.787	90.346	1.885	A	0.868

1.6. Table S6. Leave-some-out validation test for DF_1 by applying the criteria of leaving the 25% of the data set out as test set.

**L-S-O nº**	**λ**	**F**	**p <**	**Test set**	
				**JAK1 inhibitor**	**Percentage of Correct Class. (%)**
1	0.358	17.038	0.0001	Cerdulatinib- Momelotinib- Tofacitinib- BMS-911543- FLLL32- JANEX-1- WHI-P154	71
2	0.401	14.220	0.0002	Baricitinib- Cerdulatinib- Tofacitinib - BMS-911543- Go6976- Pacritinib- WHI-P154	86
3	0.434	12.391	0.0004	Filgotinib- Oclacitinib- ZM39923hydrochloride- CEP33779- Gandotinib- NVP-BSK805- WHI-P97	71
4	0.525	8.585	0.0022	Baricitinib- Itacitinib- Solcitinib- AZD1480- Decernotinib- Hexabromocyclohexane- TG101209	86
5	0.418	13.231	0.0003	Momelotinib- PF-04965842- Solcitinib- Tofacitinib- BMS-911543- FLLL32- JANEX-1	71
6	0.413	13.480	0.0002	Cerdulatinib- Momelotinib- Tofacitinib- Decernotinib- NVP-BSK805- WHI-P154- XL019	86
7	0.477	10.407	0.0009	Momelotinib- Oclacitinib- AZD1480- FLLL32- Gandotinib- NVP-BSK805- WHI-P154	86
8	0.374	15.891	0.0001	Filgotinib- Solcitinib- ZM39923hydrochloride- BMS-911543- Gandotinib- JANEX-1- WHI-P97	57
9	0.548	7.836	0.0033	Cerdulatinib- Itacitinib- Oclacitinib- Solcitinib- AT9283- Decernotinib- NVP-BSK805	100
10	0.352	17.462	0.00001	Momelotinib- Ruxolitinib- ZM39923hydrochloride- BMS-911543- Decernotinib- WHI-P154- WP1066	57
**Average**	0.358	13.054	0.0009		77
**DF_2**	0.462	15.155	0.00001		

1.7. Table S7. Leave-some-out validation test for DF_2 by applying the criteria of leaving the 25% of the data set out as test set.

L-S-O nº	λ	F	p <	Test set: JAK2 inhibitors	Test set: Percentage of Correct Class. (%)
1	0.547	5.255	0.0083	FLLL32- Hexabromocyclohexane- TG101209- JANEX-1- Peficitinib- WHI-P97-	100
2	0.462	7.384	0.0018	AZ960- BMS-911543- Cerdulatinib- XL019- Itacitinib- PF 06551600malonate- ZM39923hydrochloride	86
3	0.473	7.066	0.0022	Baricitinib- Go6976- Pacritinib- TG101209- Itacitinib- Peficitinib-	86
4	0.402	9.407	0.0005	Cerdulatinib- Filgotinib- NVP-BSK805- Pacritinib- Decernotinib- Itacitinib- PF 06551600malonate-	71
5	0.459	7.474	0.0017	AZ960- CEP33779- FLLL32- NVP-BSK805- WP1066- WHI-P154- ZM39923hydrochloride	57
6	0.479	6.895	0.0025	BMS-911543- Filgotinib- Momelotinib- TG101209- Itacitinib- WHI-P154	86
7	0.505	6.198	0.0041	Baricitinib- Gandotinib- Ruxolitinib- AZD1480- PF 06551600malonate- WHI-P154- WHI-P97-	86
8	0.397	9.604	0.0005	AZ960- BMS-911543- Filgotinib- Go6976- PF-04965842- XL019- JANEX-1-	86
9	0.512	6.026	0.0046	Filgotinib- Hexabromocyclohexane- Momelotinib- Pacritinib- TG101209- Decernotinib- PF 06551600malonate-	86
10	0.485	6.729	0.0028	CEP33779- Go6976- NVP-BSK805- Pacritinib- XL019- AZD1480- JANEX-1-	86
**Average**	**0.472**	**7.204**	**0.0029**		**83**
**DF_3**	**0.490**	**9.009**	**0.0003**		

1.8. Table S8. Leave-some-out validation test for DF_3 by applying the criteria of leaving the 25% of the data set out as test set.

L-S-O nº	λ	F	p <	Test set: JAK3 inhibitors	Test set: Percentage of Correct Class. (%)
1	0.464	4.614	0.011	AT9283 – Filgotinib-WHI-P154-AZ-960-FLLL32-Itacitinib-Pacritinib	100
2	0.500	3.999	0.020	PF 06551600malonate-WHI-P97-BMS-911543-Go6976-NVP-BSK805-PF-04965842-XL019	100
3	0.359	7.151	0.002	Cerdulatinib -JANEX-1-Tofacitinib-ZM39923hydrochloride-BMS-911543-Hexabromocyclohexane-Oclacitinib	57
4	0.480	4.335	0.015	AT9283 - AZD1480-JANEX-1-WHI-P97-CEP33779-Itacitinib-PF-04965842	86
5	0.381	6.503	0.003	Decernotinib-Tofacitinib-AZ-960-FLLL32-Itacitinib-Oclacitinib-TG101209	86
6	0.485	4.250	0.016	PF 06551600malonate-WHI-P97-BMS-911543-Momelotinib-NVP-BSK805-PF-04965842-WP1066	100
7	0.470	4.509	0.013	AT9283 - Decernotinib-Tofacitinib-WHI-P97-Baricitinib-Gandotinib-Momelotinib	86
8	0.433	5.243	0.007	AZD1480-Cerdulatinib-PF 06551600malonate-ZM39923hydrochloride-FLLL32-Hexabromocyclohexane-Pacritinib	71
9	0.491	4.141	0.017	Filgotinib-WHI-P154-Baricitinib-FLLL32-Itacitinib-Oclacitinib-TG101209	86
10	0.449	4.914	0.009	AZD1480 - JANEX-1-WHI-P97-Baricitinib-Gandotinib-Momelotinib-WP1066	100
**Average**	0.451	4.966	0.011		87
**DF_4**	0.462	6.706	0.001		

1.9. Table S9. Docking score values from potential JAK inhibitors selected by Molecular Topology.

Potential JAK inhibitors	Docking score JAK1	Docking score JAK2	Docking score JAK3	Selected
**AA-516-0011028**	-7.06	-7.61	-8.00	*
AC-907-4131030	-7.82	-7.53	-8.11	*
AE-848-4779061	-6.67	-7.20	-9.02	*
AF-399-37297037	-7.38	-7.44	-7.75	*
AF-399-41945530	-6.82	-8.36	-6.72	*
**AF-399-2100326**	-6.82	-9.46	-6.96	*
AF-399-42762404	-8.51	-7.45	-7.39	*
**AG-205-2010072**	-8.24	-7.31	-7.89	*
**AG-670-3619018**	-6.81	-8.17	-7.26	*
**AK-778-3206447**	-9.05	-7.11	-6.90	*
AN-584-0652663	-7.44	-7.53	-7.13	*
AN-988-1531688	-6.51	-7.70	-6.53	*
AO-476-3250150	-7.05	-6.69	-7.71	*
**AO-476-3417077**	-8.14	-9.03	-8.32	*
AB-323-3887443	-5.28	-5.87	-7.33	*
AF-399-13277002	-5.86	-6.55	-7.03	*
AF-399-13426006	-5.77	-7.07	-6.58	*
AF-399-15032375	-6.09	-7.33	-7.68	*
AF-399-41668884	-5.43	-7.77	-5.14	*
**AO-476-1610187**	-5.34	-7.34	-6.88	*
AO-476-3250160	-5.79	-6.07	-7.06	*
**AQ-405-2300191**	-8.13	-6.28	-5.73	*
AQ-432-3400219	-7.12	-6.13	-7.06	*
AF-399-15031149	-5.32	-5.31	-5.91	
AF-399-33696009	-5.13	-6.65	-6.45	
AF-399-42056988	-6.76	-6.75	-6.88	
AG-205-1444099	-5.59	-5.75	-6.03	
AG-205-1674118	-6.50	-6.64	-6.79	
AG-205-4250132	-5.86	-6.09	-5.94	
AG-205-4673025	-5.79	-5.07	-6.17	
AG-401-2041003	-6.39	-5.78	-6.76	
AG-690-6926024	-5.48	-6.16	-6.90	
AH-357-3329001	-5.40	-6.26	-6.77	
AK-968-5359231	-6.67	-5.81	-6.72	
AM-807-7225018	-6.57	-6.07	-6.74	
AN-329-1658808	-4.47	-5.35	-5.26	

AN-329-1717385	-4.97	-3.15	-3.42
AN-584-3492329	-6.31	-6.37	-6.72
AO-365-3473564	-6.02	-5.70	-6.52
AO-476-3250148	-6.06	-5.84	-6.98
AP-064-42049177	-6.83	-6.35	-6.84
AP-501-43286814	-4.88	-6.52	-6.44
AQ-432-3399984	-6.87	-6.05	-6.29
AQ-432-3400108	-6.22	-6.29	-6.11
AQ-432-3400304	-6.10	-6.36	-6.25
AQ-432-3400319	-3.35	-4.00	-5.03
AT-417-3503979	-5.34	-5.31	-5.14

Bold: *in vitro* tested

2. Cross-docking analysis: results

As reported in the manuscript the cross-docking study carried out with all the crystallographic structures available in April 2019 in the PDB for JAK1, JAK2 and JAK3, identify 4ivd, 5cf6 and 6gla as the ones with the greatest percentage of ligands docked within an RMSD < 2.5 Å (blue color). It also presents a lower global value for all the complexes calculated (data not shown).

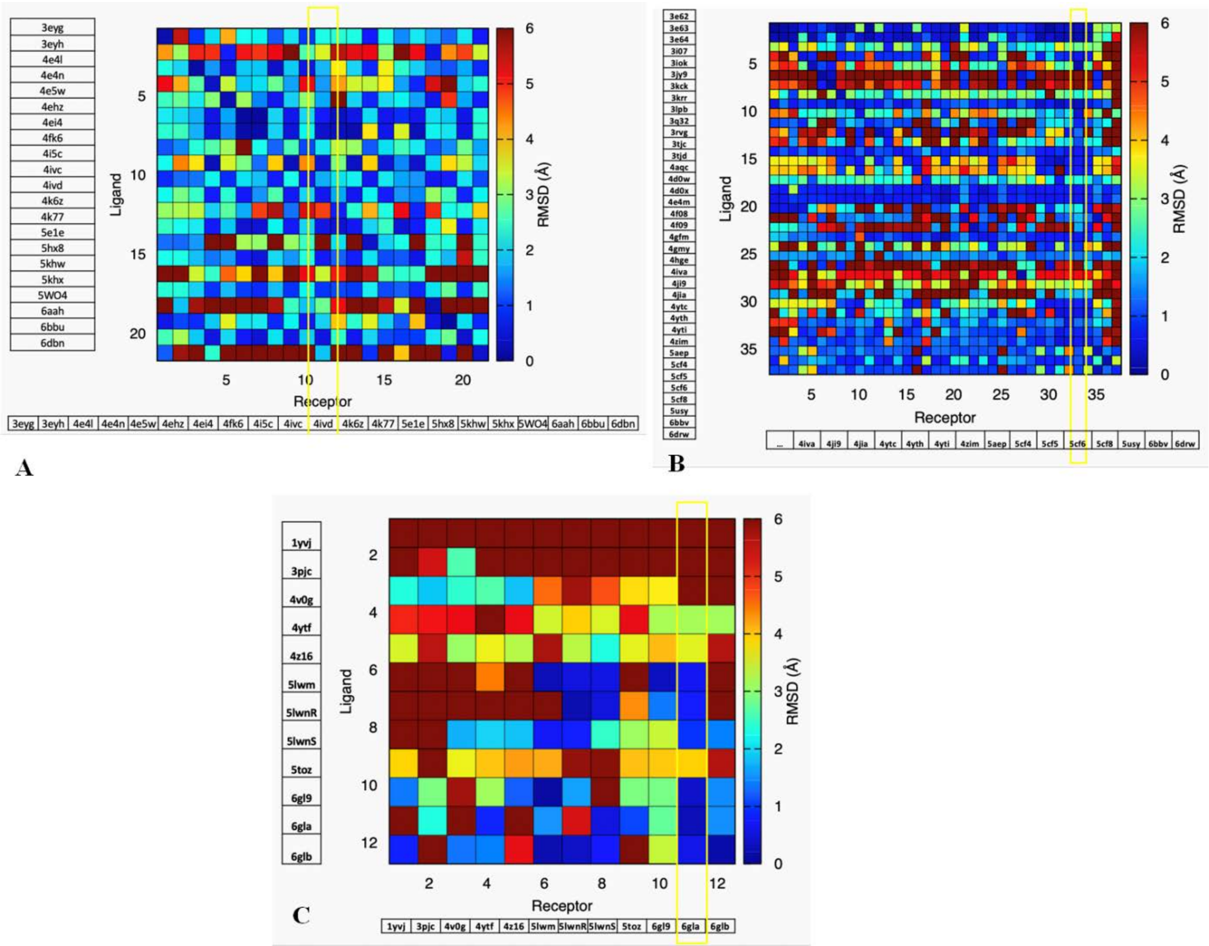

Fig.S1. Cross-docking analysis for JAK1 (A), JAK2 (B) and JAK3 (C) subtypes. Blue color: ligands docked within an RMSD < 2.5 Å.

3. Similarity-cluster analysis: Fig S2.

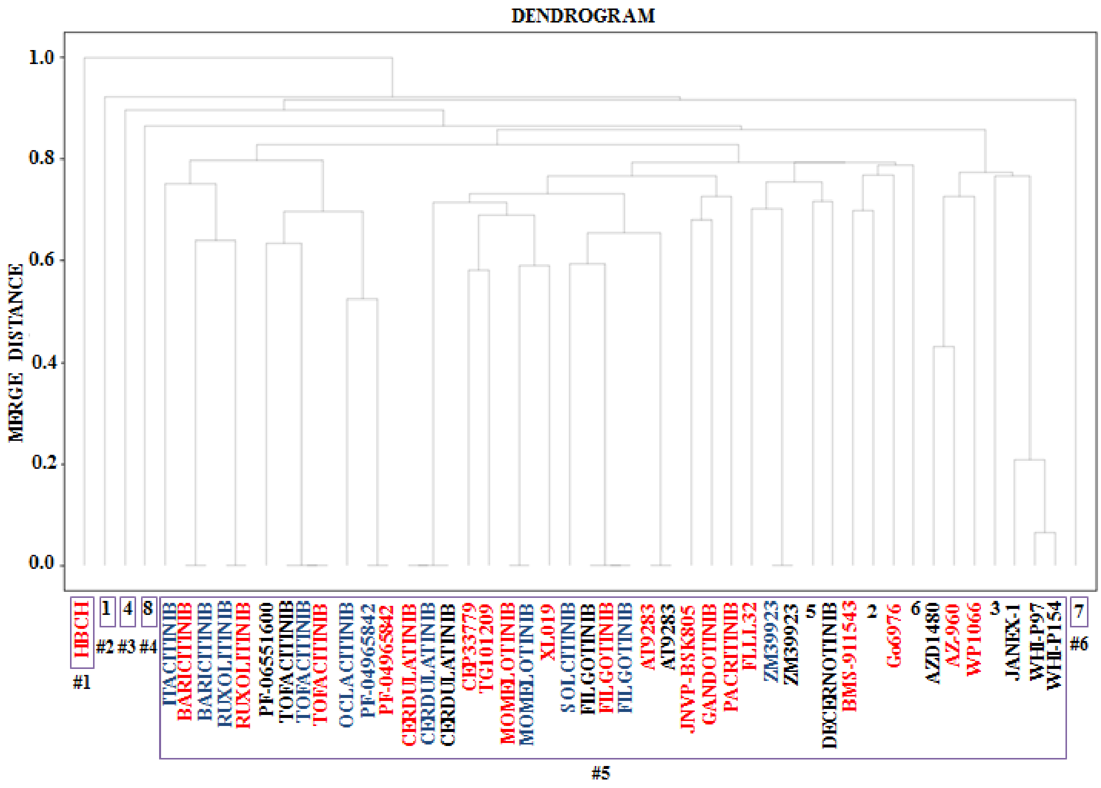

Fig.S2. Structure-similarity dendrogram (obtained with Canvas 3.9) of known and potential JAK inhibitors. Six clusters were obtained: cluster #1 (HBCH, Hexabromocyclohexane) , cluster #2 (**1**), cluster #3 (**4**), cluster #4 (**8**), cluster #5 (45 molecules, known JAK inhibitors and compounds **2**, **3**, **5** and **6**) and cluster #6 (**7**).
